# Health workforce needs in Malawi: analysis of the Thanzi La Onse integrated epidemiological model of care

**DOI:** 10.1186/s12960-024-00949-2

**Published:** 2024-09-27

**Authors:** Bingling She, Tara D. Mangal, Margaret L. Prust, Stephanie Heung, Martin Chalkley, Tim Colbourn, Joseph H. Collins, Matthew M. Graham, Britta Jewell, Purava Joshi, Ines Li Lin, Emmanuel Mnjowe, Sakshi Mohan, Margherita Molaro, Andrew N. Phillips, Paul Revill, Robert Manning Smith, Asif U. Tamuri, Pakwanja D. Twea, Gerald Manthalu, Joseph Mfutso-Bengo, Timothy B. Hallett

**Affiliations:** 1 Department of Infectious Disease Epidemiology, School of Public Health, Imperial College London, London, UK; 2 Clinton Health Access Initiative, Inc., Boston, USA; 3 Clinton Health Access Initiative, Inc., Lilongwe, Malawi; 4 Centre for Health Economics, University of York, York, UK; 5 Institute for Global Health, University College London, London, UK; 6 UCL Centre for Advanced Research, University College London, London, UK; 7 College of Medicine, University of Malawi, Lilongwe, Malawi; 8 Department of Genetics, Evolution and Environment, University College London, London, UK; 9 Department of Planning and Policy Development, Ministry of Health, Lilongwe, Malawi; 10 School of Public Health-College of Medicine, University of Malawi, Blantyre, Malawi

**Keywords:** Model design, Healthcare workforce, Health care needs, Health services, Health system interactions

## Abstract

**Background:**

To make the best use of health resources, it is crucial to understand the healthcare needs of a population—including how needs will evolve and respond to changing epidemiological context and patient behaviour—and how this compares to the capabilities to deliver healthcare with the existing workforce. Existing approaches to planning either rely on using observed healthcare demand from a fixed historical period or using models to estimate healthcare needs within a narrow domain (e.g., a specific disease area or health programme). A new data-grounded modelling method is proposed by which healthcare needs and the capabilities of the healthcare workforce can be compared and analysed under a range of scenarios: in particular, when there is much greater propensity for healthcare seeking.

**Methods:**

A model representation of the healthcare workforce, one that formalises how the time of the different cadres is drawn into the provision of units of healthcare, was integrated with an individual-based epidemiological model—the *Thanzi La Onse* model—that represents mechanistically the development of disease and ill-health and patients’ healthcare seeking behaviour. The model was applied in Malawi using routinely available data and the estimates of the volume of health service delivered were tested against officially recorded data. Model estimates of the “time needed” and “time available” for each cadre were compared under different assumptions for whether vacant (or established) posts are filled and healthcare seeking behaviour.

**Results:**

The model estimates of volume of each type of service delivered were in good agreement with the available data. The “time needed” for the healthcare workforce greatly exceeded the “time available” (overall by 1.82-fold), especially for pharmacists (6.37-fold) and clinicians (2.83-fold). This discrepancy would be largely mitigated if all vacant posts were filled, but the large discrepancy would remain for pharmacists (2.49-fold). However, if all of those becoming ill did seek care immediately, the “time needed” would increase dramatically and exceed “time supply” (2.11-fold for nurses and midwives, 5.60-fold for clinicians, 9.98-fold for pharmacists) even when there were no vacant positions.

**Conclusions:**

The results suggest that services are being delivered in less time on average than they should be, or that healthcare workers are working more time than contracted, or a combination of the two. Moreover, the analysis shows that the healthcare system could become overwhelmed if patients were more likely to seek care. It is not yet known what the health consequences of such changes would be but this new model provides—for the first time—a means to examine such questions.

**Supplementary Information:**

The online version contains supplementary material available at 10.1186/s12960-024-00949-2.

## Introduction

There is growing consensus of the need to study and invest in the healthcare system infrastructure of human and capital resources on which delivery of specific healthcare and public health interventions depends [1]. One crucial question that Ministry of Health planning departments are faced with is how many and what types of healthcare workers are needed to meet the vast and varied healthcare needs of the populations they serve. One eminent example of a government tackling this question is the Malawi Human Resources for Health Strategic Plan [2, 3]. This uses the “Workforce Optimisation Model” (WFOM) to describe the healthcare workforce staffing that would have been required to meet fully the health service delivery volume observed in a particular year [3]. Another example is the “Workload Indicators of Staffing Need (WISN)” method that has been notably used in low and middle-income countries to estimate health workforce needs based on current workload [4–7]. Such analyses, which are mostly based on existing patterns of service use, make a crucial contribution to planning.

However, such analyses do not show how the staffing requirements would change over time in response to changes in patients’ healthcare seeking behaviour, shifts in the epidemiological context, or demographic dynamics. In fact, this points to a deeper issue in analyses of the healthcare workforce: by relying only on observed data (i.e. demand for current or target levels of health service utilisation), the effect of secular changes in epidemiological context or behaviour cannot be directly measured or taken into account [8, 9].

For that reason, modelling has been turned to as a means of answering questions in healthcare workforce planning. In models, the need for healthcare can be linked to the epidemiological and behavioural processes in the population, and parameters can be manipulated to proxy the effects of future changes. Although there are many models that represent the epidemiology of a disease and the need for particular types of services, existing representations have been limited to: (i) describing the workforce abstractly with no connection to the epidemiological and behavioural drivers of healthcare needs or statically with no examination of the consequences of receiving care (or not) [10–12]; (ii) analysing workforce on sub-national and regional levels instead of the national level considering the holistic and complex health system and the connections between fundamental components including delivery platforms, services, workforce and population [9, 13, 14]; (iii) representing the constraints on healthcare worker time indirectly (e.g., as a fixed “cost per appointment” or via a “cost-effectiveness threshold”) [15]; (iv) representing healthcare worker time of a uniform type being divided into “slots” that are allocated and can be exhausted [16, 17], and (v) typically focusing on single diseases or vertical programmes in isolation [13–15, 18–20]. As a result, there have been repeated calls for models that span all health conditions of interest (e.g., diseases, injuries, maternity and delivery), including the feedback, long-term dynamics and the impacts of demographic changes, married with a detailed representation of the whole national healthcare system, especially for low and middle-income countries [6, 9, 14, 21, 22].

The Thanzi La Onse (TLO) Model was developed to meet that need [23, 24]. The epidemiological components of the framework have been described elsewhere [23]. Here, the representation of the health workforce capabilities in the model are described, and analyses are presented showing the estimated needs of health workforce in Malawi under various assumptions for recruitment of healthcare workers and patient healthcare seeking behaviour.

## Methods

First, the approach for modelling the healthcare workforce capability is described. Second, the data sources for health workforce and health service utilisation in Malawi are described. Third, the ways in which the model is applied in order to estimate how healthcare worker time is used currently and how this could change under alternative scenarios are described.

### Model design of the health workforce capability in the TLO model

**Fig. 1:**
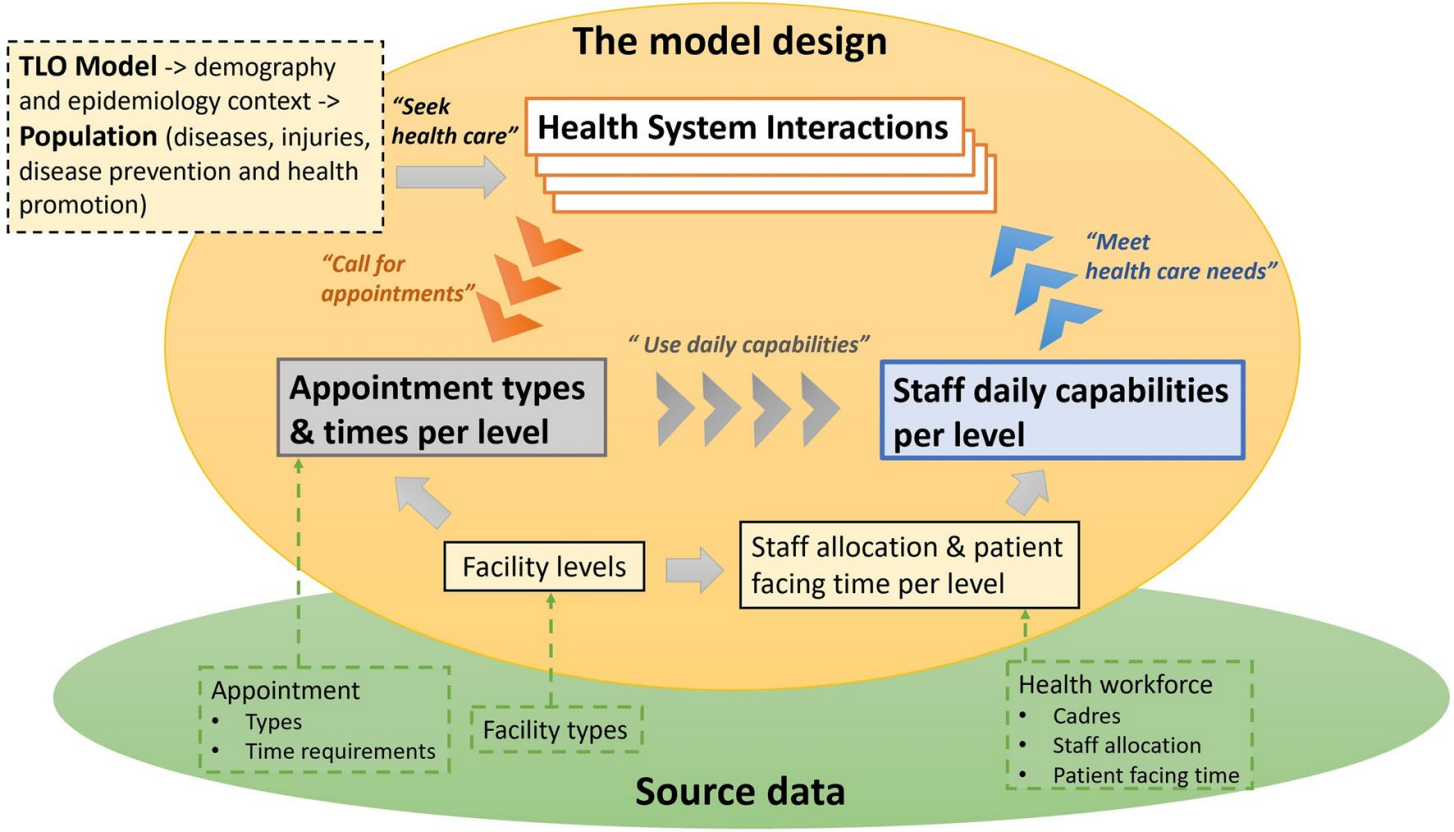
Model design for healthcare workforce capability

Figure 1 and Box 1 illustrate the model structure and describe each key concept in detail, respectively, and Box 2 summarises the TLO model. The code that represents this model framework is available at TLO repository [25]. Briefly, in this model framework, any care received by individuals from the healthcare system is encapsulated in a “Health System Interaction” (HSI) event. Each HSI occurs at a particular “Facility Level” and declares the time required of the health workforce in the form of a set of standardised “Appointment Types”. Each “Appointment Type” consumes a fixed amount of time of each healthcare worker cadre. The “Daily Capability” of each cadre at each level to provide services for these HSIs is the total amount of “Patient Facing Time” of staff of that cadre at that level each day.

#### Box 1. Summary of the key concepts used in the model


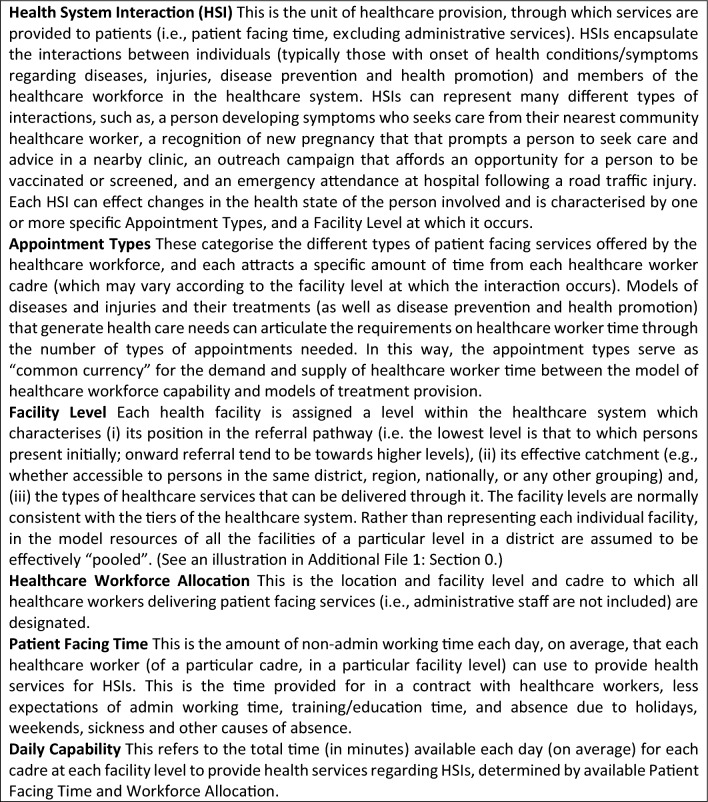


#### Box 2. The Thanzi La Onse Model


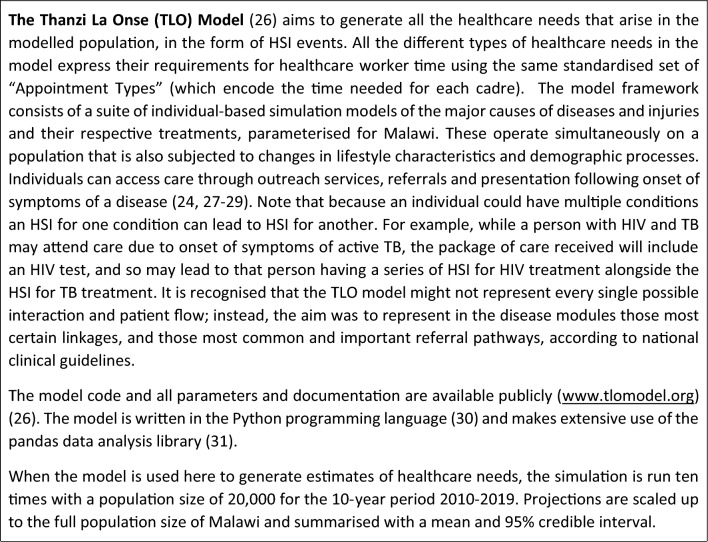


As all the different types of healthcare services in the model can be written in terms of the same set of appointment types, it represents coherently the provision of diverse sets of care for different disease and conditions. In addition, as the time of healthcare workers can be drawn into different forms of appointments, this framework captures the workflow and flexibility of workers to respond to the health care needs with which they are faced.

### Data sources for the healthcare workforce and health services utilisation in Malawi

The model was parameterised using data from the Detailed Annex for the Health Workforce Interventions of the Malawi Health Sector Strategic Plan (HSSP III) for 2023–2030 (HSSP III HRH Annex) [32]. This data source provides comprehensive information on Malawi healthcare system and healthcare workforce in public sector and the Christian Health Association of Malawi (CHAM), including: health facility types, health service appointments and time requirements at major facility types, actual and established healthcare workforce counts by cadre and by district and central hospital (established positions refer to positions that are targeted to be filled [3]), and the available working time for cadres at hospitals and health centres.

For validating the model, the volume of health services delivered in the model is compared with those reported in the Malawi health management information systems [33, 34].

### Analysis

In Part I, the fundamental components in the healthcare workforce capability model for Malawi are defined using the available data: i.e. facility levels, healthcare cadres and patient facing times, and appointment types. On the basis of information on the actual and established staff counts, there are two scenarios for the healthcare workforce allocation: the ‘Actual Scenario’ and the ‘Establishment Scenario’. The resulting estimated daily capabilities of each cadre at each facility level and district are presented. The health system interactions at one facility level is also illustrated.

In Part II, this representation of the workforce in the model is used to estimate the usage of healthcare workforce capabilities when it is confronted with the healthcare needs generated in the TLO model. This analysis is repeated for each of the Actual and Establishment Scenarios. Finally, the analysis is re-run when the assumption of healthcare seeking is updated so that all persons seek care promptly upon symptom onset of any disease or condition (i.e. from the ‘Default’ that reflects current healthcare seeking to the ‘Maximal’ healthcare seeking).

## Results

### Part I: definitions of workforce model components

#### Facility levels

Table 1 presents the facility levels defined for Malawi healthcare system, including local level 0, primary levels 1a and 1b, secondary level 2, tertiary level 3, and national levels 4 and 5.

**Table 1:** Facility-level representation

Facility level	Description	Facility type	Facility quantity
0	Facilities provide service at local level and have little infrastructure	Health Post, Village Health Committee, Community Health Station, Village/Mobile/Outreach Clinic, and other community initiatives	One set per district
1a	Facilities provide services at primary level but have less capacity than hospitals	Dispensary, Rural/Urban Health Centre, Private/Special/Antenatal Clinic, Maternity Clinic/Facility	One set per district
1b	Hospitals provide services at primary level	Community/Rural Hospital, CHAM (community) Hospital	One set per district
2	Facilities provide services at district level and provide referral services to primary level facilities	District Hospital, District Health Office*	One set per district
3	Central hospitals provide specialist health services at regional level and provide referral services to district-level facilities in that region	Kamuzu Central Hospital (KCH, Central), Mzuzu Central Hospital (MCH, North), Zomba Central Hospital (ZCH, South) & Queen Elizabeth Central Hospital (QECH, South)	One set per region
4	National level resource hospitals and centres	Zomba Mental Hospital (ZMH)	One set
5	Ministry of Health Headquarters	Headquarters*	One set

Each facility level is given a description, a set of relevant facility types as collected from elsewhere [2, 5, 10, 11, 32, 33], and the facility quantity. Each district has its own sets of level 0, level 1a, level 1b and level 2 facilities; each region (Northern, Central, Southern) has its own set of level 3 facilities (central hospitals), called ‘Referral Hospital—Northern/Central/Southern’ to differentiate with ‘central hospital’ as southern region has two central hospitals; on the national level, there are Zomba Mental Hospital and Headquarters. Therefore, Malawi has 133 (= 4 × 32 + 5) facility sets in total (including headquarters that do not deliver patient facing services directly though), where the 32 districts from 2018 Malawi Census [35] include 27 general districts and 5 special districts as specified in Additional file 1: Sect. 1. *District Health Offices and Headquarters are administrative health institutions that do not provide patient facing services directly

#### Healthcare cadres and patient facing time

Table 2 lists the 21 healthcare cadres and 9 categories that are recognised in the Malawi healthcare system. The patient facing time per cadre at each facility level is detailed in Additional file 1: Sect.  2.

**Table 2:** Healthcare cadres and categories

Cadre category	Cadre
Clinical	M01 Medical Officer/Specialist, M02 Clinical Officer/Technician, M03 Medical Assistant
Nursing and Midwifery	N01 Nurse Officer, N02 Nurse Midwife Technician
Pharmacy	P01 Pharmacist, P02 Pharm Technician, P03 Pharm Assistant
Laboratory	L01 Lab Officer, L02 Lab Technician, L03 Lab Assistant
DCSA/HSA	E01 DCSA/HSA (Disease Control and Surveillance Assistant/Health Surveillance Assistant)
Dental	D01 Dental Officer, D02 Dental Therapist, D03 Dental Assistant
Mental	C01 Mental Health Staff
Nutrition	T01 Nutrition Staff
Radiography	R01 Radiographer, R02 Radiography Technician, R03 Sonographer, R04 Radiotherapy Technician

The cadres and cadre categories are defined by the data source [32]. Note there are other cadres not included due to low relevance to patient facing services, e.g., administrative staff

#### Appointment types

Table 3 lists 11 appointment categories and 49 appointment types. See Additional file 1: Sect.  3 for full descriptions for each Appointment Type.

**Table 3:** Appointment categories and types

Appointment category	Appointment type
IPOP	InpatientDays, IPAdmission (Inpatient admission and discharge), Under5OPD (Outpatient visit for under 5 years old), Over5OPD
RMNCH	NormalDelivery, CompDelivery (Complicated delivery), Csection, FamPlan (Family Planning), AntenatalFirst (Antenatal care first visit), ANCSubsequent (Antenatal care follow-up visit), EPI (Vaccination in Expanded Programme on Immunisation), STI (Sexually transmitted infections treatment)
NUTRITION	U5Malnutr (Malnutrion treatment for under 5 years old)
MISC	AccidentsandEmerg, MajorSurg (Major surgery), MinorSurg
TB	TBNew (TB first visit), TBFollowUp (TB follow-up visit)
HIV	VCTNegative (HIV test negative), VCTPositive, MaleCirc (Male circumcision), NewAdult (on ART), EstMedCom (Established medically complex adult on ART), EstNonCom (Established non-medically complex adult on ART), PMTCT (Pregnant female on ART), Peds (Pediatrics on ART)
LABORATORY	LabHaem, LabPOC, LabParasit, LabBiochem, LabMicrobio, LabMolec, LabTBMicro, LabSero, LabCyto, LabTrans
RADIOGRAPHY	Ultrasound, Mammography, MRI, Tomography, Radiotherapy, DiagRadio
DENTAL	DentAccidEmerg (Dental accidents and emergency visit), DentSurg (Dental surgery), DentalU5(Dental outpatient visit for under 5 years old), DentalO5
MENTAL	MentOPD (Mental outpatient visit), MentClinic (Mental clinic visit)
ConWithDCSA	ConWithDCSA (Health Consultation with DCSA)

The selection of appointment categories and types follows that used by the HSSP III HRH Annex, which are based on Malawi's Health Benefits Package [32]

**Fig. 2:**
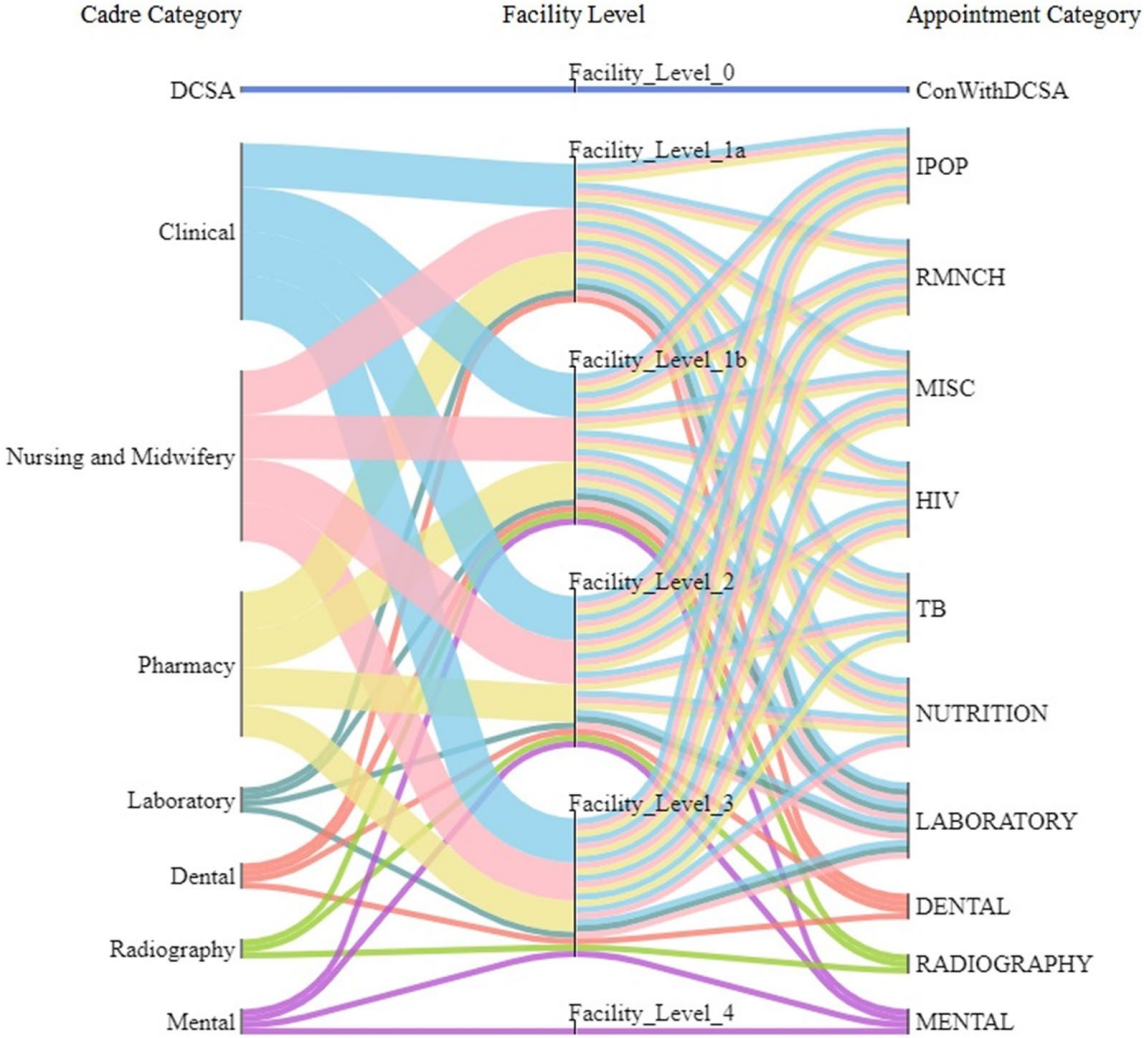
Mapping cadre category and appointment category according to appointment time requirement. This diagram demonstrates the required health worker cadres to deliver each health service type at each facility level. For example, the top flow shows that DCSA cadre is required to deliver the health service of consultancy with DCSA at level 0. Note that Nutrition cadre category is not presented because of no appointment requiring their working time in the Data Source. These Sankey diagrams were plotted using the FLOWEAVER package [36]

For each Appointment Type at each level, the average time requirement for each healthcare cadre is calculated using data for different facility types including central/district/community hospitals and urban/rural health centres. (See Additional file 1: Sect. 3 for a full description of the definitions and assumptions made for appointment time requirements per level.) Figure 2 illustrates the mapping between cadre categories and appointment categories at each level. Clinical, Nursing and Midwifery, and Pharmacy cadres are needed by many appointment types at primary, secondary and tertiary levels. By contrast, Dental, Laboratory, Mental and Radiography cadres are mostly called by their respective specialist appointment categories. As expected, the DCSA cadre is only called by “ConWithDCSA” appointment at level 0. At level 4, only the Mental cadre is called to deliver mental health appointments: this is a specialist mental hospital. Figures in Additional file 1: Sect. 4 further map the cadre categories to appointment types at each level.

#### Workforce allocation and daily capabilities

**Fig. 3:**
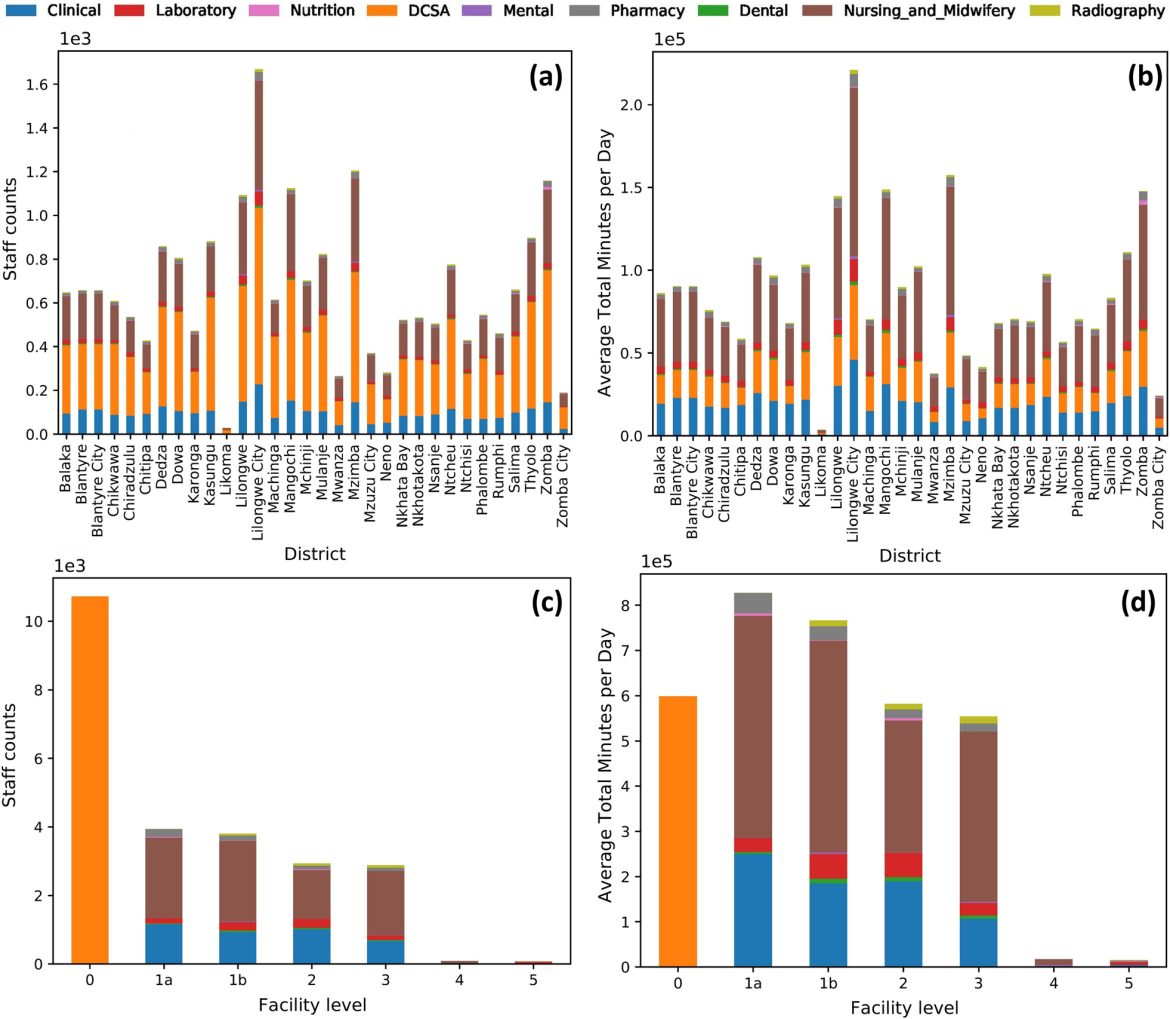
Actual scenario workforce allocation and daily capabilities. **a** Staff counts per cadre category by district, **b** daily minutes available per cadre category by district, **c** staff counts per cadre category by level, **d** daily minutes available per cadre category by level. See the Actual Scenario workforce allocation and daily capabilities per district-level in Additional file 1: Sect. 5

Figure 3 presents the healthcare workforce allocation and daily capabilities in the Actual Scenario (wherein only staff actually employed are included). The Clinical, Nursing and Midwifery, and DCSA cadres have the greatest daily capabilities. The Facility Level 0 has the greatest number of staff (DCSAs), which is more than twice that of other levels. However, since the patient facing time of DCSA cadre is much less than other cadres (see Additional file 1: Sect. 2), there is less difference in the total amount of patient facing time between the levels. The corresponding results for the Establishment Scenario (wherein all funded positions are included, including those currently vacant) are presented in Additional file 1: Sect. 6 and Sect. 7.

#### Health system interactions

**Fig. 4:**
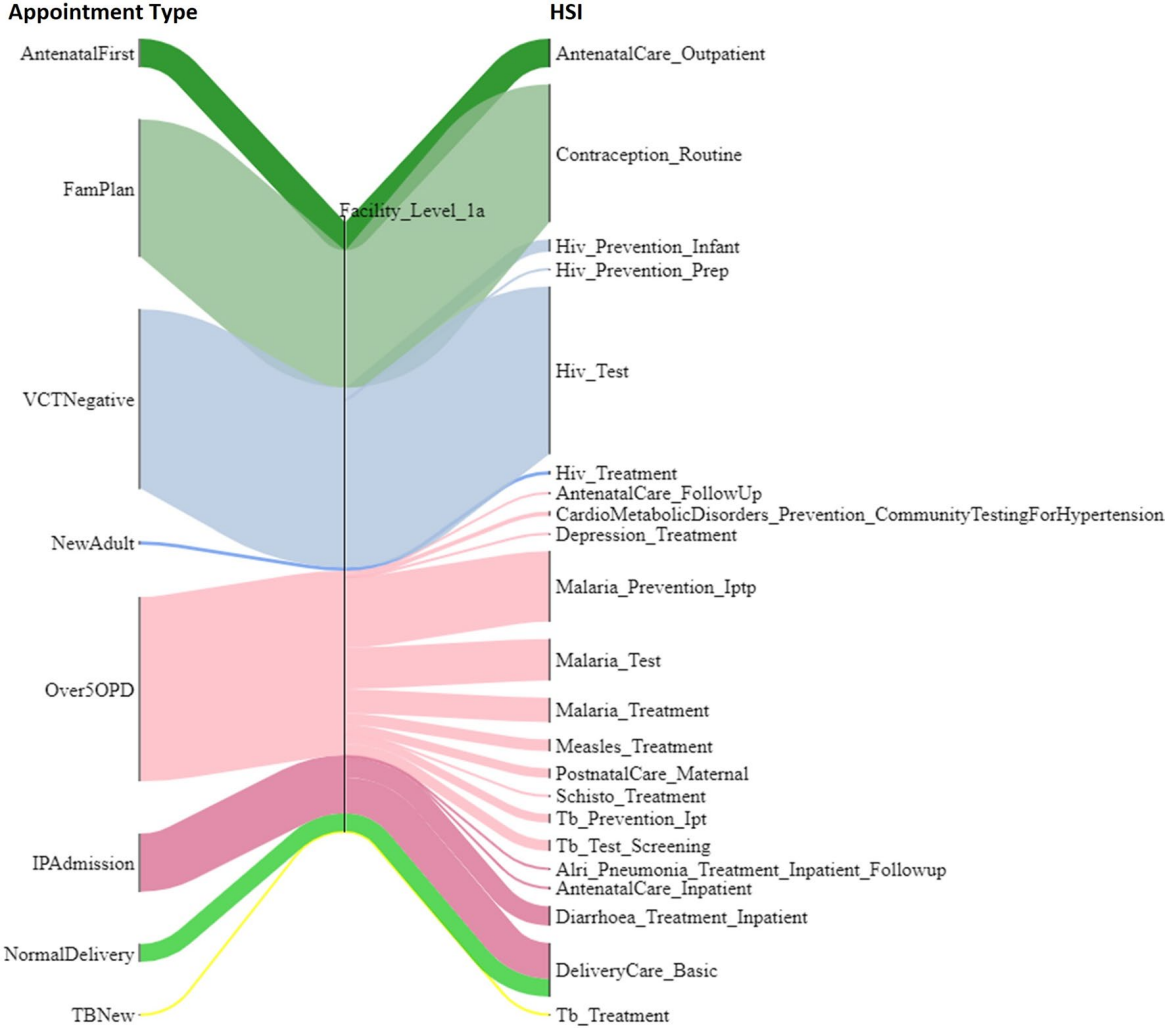
Illustration of exemplar appointment types being drawn upon by HSIs at facility level 1a. The HSIs on the right are named in the format of “Disease module_Treatment type”: for example, “HIV_Test” means the health system interaction event of test for HIV. Full details of HSIs can be found in the TLO documentation [24, 37]. The width of each flow is proportional to the number of calls for each Appointment Type in the model

The healthcare workforce model can now be used within the TLO Model. Figure 4 illustrates the different HSIs that represent care being provided for different conditions using the same system of appointment types at facility level 1a. The corresponding diagram of appointment types being drawn upon by HSIs at all levels is included in Additional file 1: Sect. 8. It shows that the HSIs that require “Over5OPD” appointment include those representing diagnosis and treatment for malaria, antenatal and postnatal care for women, and TB. The majority of calls for “Over5OPD” are for malaria services. As the healthcare worker time for the “Over5OPD” is finite, this shows the provisioning of one type of service in the model is in direct “competition” with others for the same healthcare worker time. Thus, when there is insufficient time for all demand to be met, the health opportunity cost can manifest automatically in the reduced provision of some service types.

### Part II: usage of health services and workforce capabilities

**Fig. 5:**
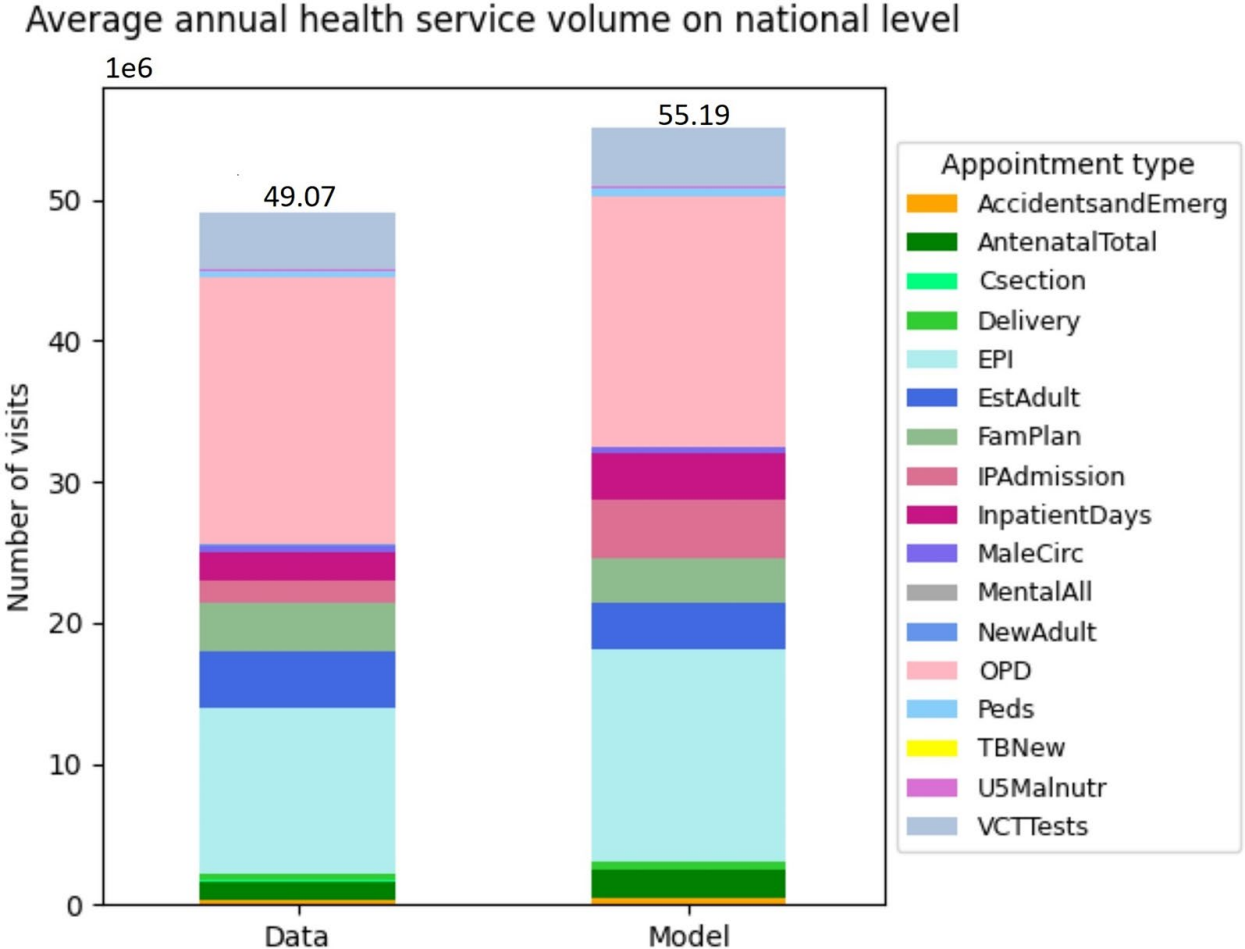
Simulated vs officially recorded annual average health service volume on national level 2015–2019. Refer to Table 3 above and the table in Additional file 1: Sect. 3 for descriptions of appointment types. The health service volume for each appointment type is measured by number of visits or cases or frequencies

Figure 5 shows a comparison between the simulated health services volume with that officially recorded in terms of annual average appointment counts/visits for years 2015–2019 on the national level. In total, the simulated volume is 55 million visits, which is 12% greater than the officially recorded value of 49 million. However, the distribution of services across the categories of care are highly comparable between the model and data, with the possible exception of “EPI” (vaccination appointments). As it is expected that the data might be an incomplete account of the services provided (e.g., the estimated reporting rate of vaccinations is 84% [34]), this is the evidence that the model and the data are in remarkably good agreement.

**Fig. 6:**
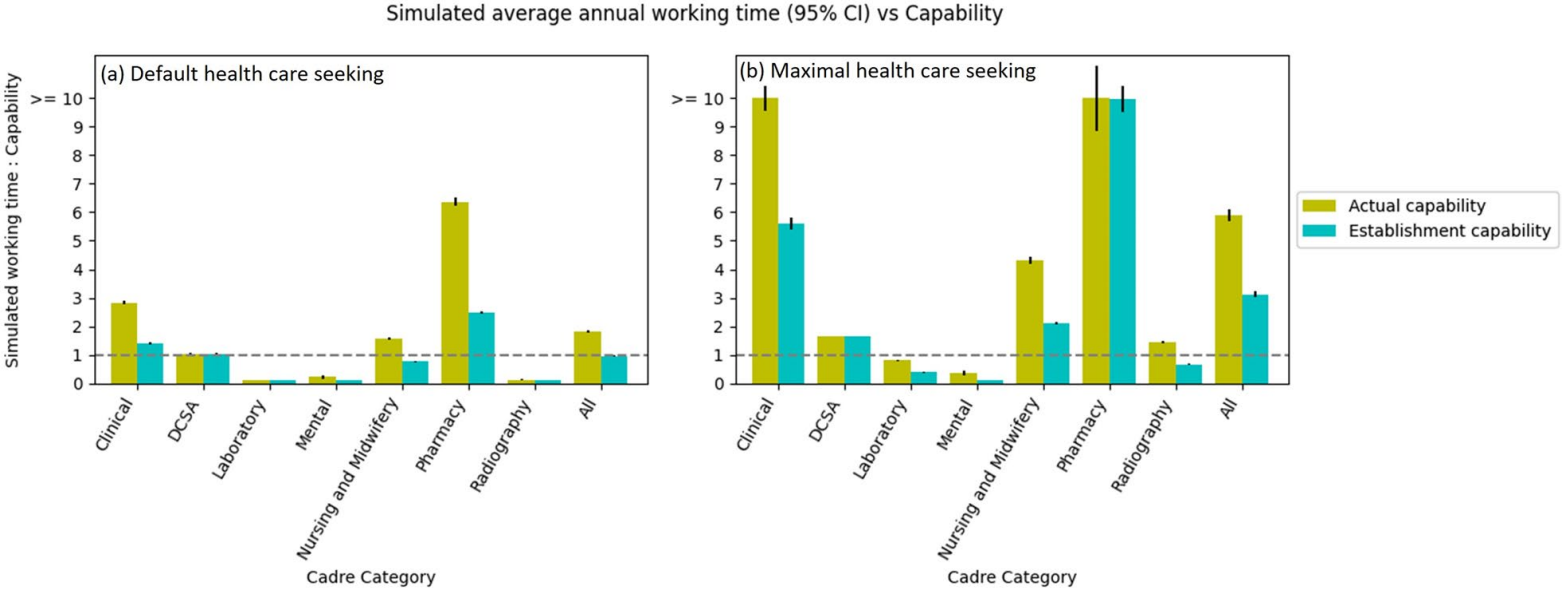
Comparison of simulated average annual working time and actual/establishment capabilities per cadre category in two health care seeking scenarios. The annual working time is the annual number of appointments times the appointment time requirements. The line of “simulated working time: capability” = 1 means the usage of healthcare workers’ patient facing time in the model perfectly matches their capabilities. Note that since the data for the ConWithDCSA appointment time requirement are not available, in the simulation, it is assumed such a value (i.e. 20 minutes for DCSA per appointment) that the simulated total working time can well match the Actual capabilities of DCSA; and that the extra capabilities of Laboratory and Radiography cadres may be due to that cancer modules in TLO Model have not fully represented the HSIs of laboratory and radiography services such as diagnostic test and screening on one hand and that the laboratory and radiography appointment time requirements might have been underestimated in data source on the other hand. Also note that Nutrition and Dental cadres are not analysed because of no relevant service data

Figure 6a shows the estimate of the ratio for the time-needed for all healthcare needs in the model to the time-available of each cadre. For the Actual Scenario, it shows that over all cadres, the total time required is 1.82 times the total actual capability. This means that it would not be possible for the services to have been delivered by the available staff without either: (i) each appointment taking less time than source data indicated, or (ii) staff working longer hours than contracted. The ratio is highest for Pharmacy (6.37 times), Clinical (2.83 times), and Nursing and Midwifery (1.57 times) cadres. By contrast, DSCA, Laboratory, Mental and Radiography carders seemingly have sufficient capabilities, although this could be partly a reflection of the model having a simplified representation of some relevant diseases (such as cancers).

In the Establishment Scenario, the time-needed to time-available ratio is much closer to parity overall, including for Clinical, Nursery and Midwifery and Pharmacy cadres. This indicates that much of the mismatch in time-available and time-needed in the Actual Scenario is due to vacancies in posts. However, even under the Establishment scenario, there is a much higher demand on the time of pharmacists, in particular (time-needed is 2.49 times of time-available).

Figure 6b shows the same statistics under the alternative scenario of maximal healthcare seeking. In this scenario there is greater demand for healthcare, as all persons seek care when they become ill with symptoms that would normally require healthcare services. Thus, this scenario reflects the demand of services that the system would ideally be suited to accommodate. In this scenario, the overall time-needed to time-available ratio would be 5.90 in the Actual Scenario and 3.11 in the Establishment Scenario, suggesting that there would be insufficient staff even if there were no post vacancies. In some cases, the mismatch is extreme: for example, the time-needed of Clinical and Pharmacy staff would exceed 10 times that available.

**Fig. 7:**
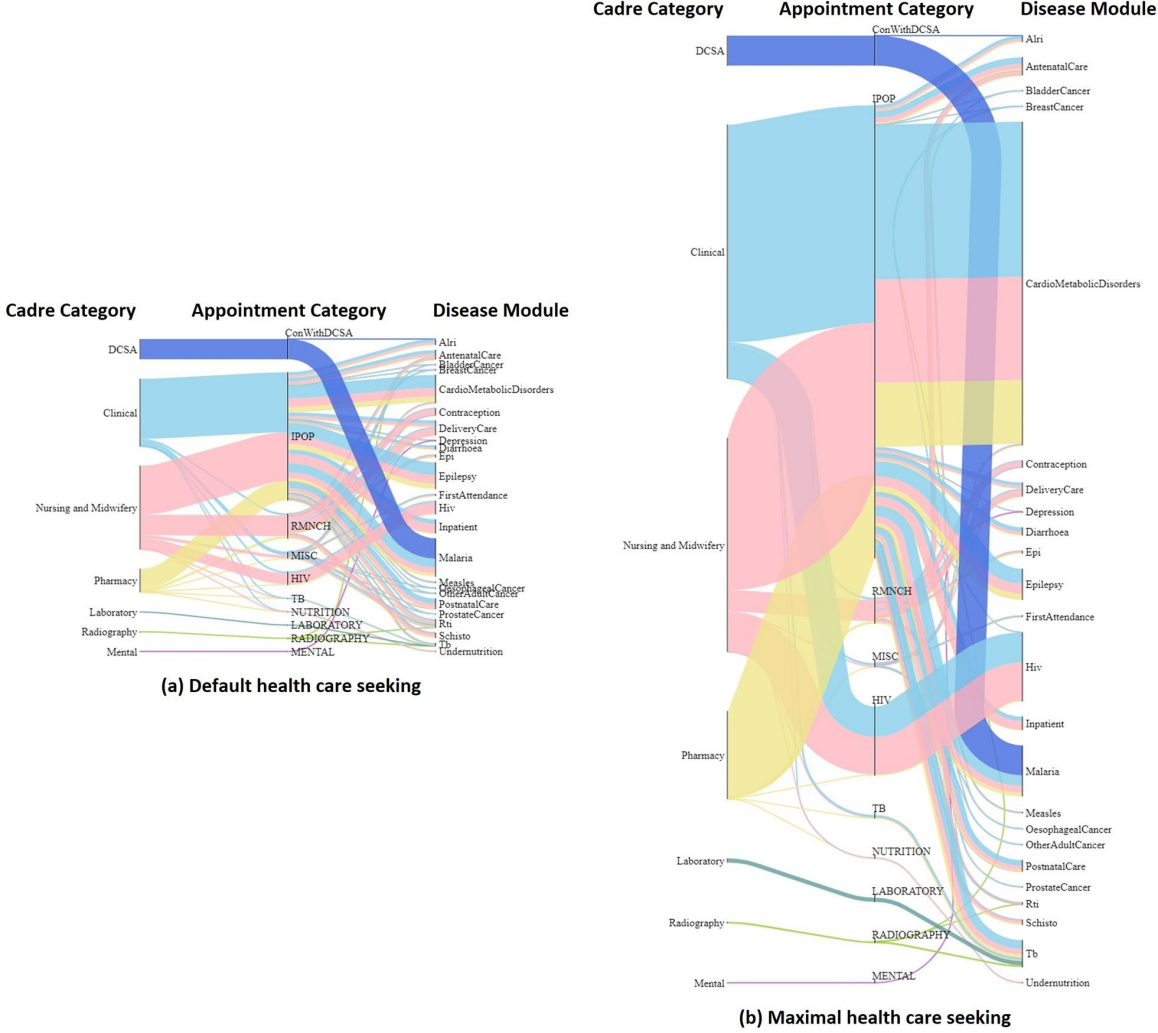
Simulated annual working time flow per cadre category in two health care seeking scenarios. The overall height of each diagram reflects the annual total working time required in each scenario, and the width of each flow reflects the proportion of working time required for each cadre by each disease module. The total working time in Maximal health care seeking scenario (7.14 × 10^9 minutes per year) is approximately 3.23 times of the Default scenario (2.21 × 10^9 minutes per year). Note that each disease module here represents a group of relevant diagnosis and treatment HSI events. Particularly, the “FirstAttendance” module represents HSI events that capture the first contact of a person seeking health care from the healthcare system, and the “Inpatient” module represents HSIs that specify the needs of inpatient care

Figure 7 compares the requirements for healthcare worker time under the two scenarios for healthcare seeking: ‘Default’ and ‘Maximal’. Many different types of treatment draw on the time of clinicians, nurses and midwives, and pharmacists, which explains why these are the most heavily demanded cadres in all cases. In the scenario with maximal healthcare seeking, the time needed increases for all types of treatment, but especially for services relating to cardio metabolic disorders and HIV. This indicates that needs for those services in particular are currently curbed by lower healthcare seeking, and that increases in those services would most contribute to the need for greater workforce capabilities.

## Discussion

A model of healthcare workforce capability has been presented and integrated into a dynamic simulation model of healthcare needs. The application of the model in Malawi shows that there are currently shortages of Clinical, Nursing and Midwifery and Pharmacy cadres compared to needs, but these may be largely mitigated if all vacant posts could be filled (although there would remain shortages of Pharmacists). However, if all persons that should seek care when they become ill did so, then the needs for health care would vastly outstrip the capabilities of the healthcare system, even if there were no vacancies.

The first of these findings agrees with earlier work that use the WFOM and WISN methods noted above [3, 5]. However, this analysis is the first to show how the workforce needs change in response to changes in healthcare needs—in this case, from the effects of changes in patients’ behaviour in seeking care. This is a key contribution of integrating a workforce model with an individual-based epidemiological and behavioural simulation model of healthcare needs.

Economic evaluation, budget allocation and planning for healthcare workforce and health services have come to rely on simulation models as a source of information for decision-making [21, 22, 38, 39]. Modelling input is helpful as this enables synthesising data on epidemiology and healthcare systems and resources to transparently provide analysis that can inform these decisions. However, earlier models have typically focussed on single diseases, isolated vertical programmes, or single technical interventions [14]. As such, these models do not allow such investments to be evaluated fully, because they do not represent how health gains are achieved through the marshalling of many different types of resources to meet a wide array of health care needs [1, 14, 15, 40, 41]. Therefore, this integrated model, which holistically represents the capabilities of the healthcare workforce and the many and varied needs it faces for health services in a healthcare system, can be used to address myriad questions that have previously eluded analysis [13, 23]. For example, (i) the effect of increasing recruitment of staff can be represented by a change in total number of staff; (ii) the effect of reallocation of staff to different facility levels can be represented by a change in the allocation of staff to each facility level; (iii) the effect of task shifting can be represented by change in appointment types (the cadres and time needed for each appointment); and, (iv) the effect of increases in productivity of workers can be represented by more minutes of patient facing time (thus less time on other activities) available per cadre. Moreover, these analyses could also be conducted in multiple scenarios of health care seeking behaviour (as investigated in this study), healthcare workforce performance and competence, and availability of other health care system resources (e.g., medical consumables and equipment) that affect the utilisation of health services and healthcare workforce capabilities [42].

A particular level of abstraction has been chosen for modelling the health workforce that is intended to accommodate answering a wide range of important questions whilst still being readily parameterisable with data that are likely to be available in many settings. As such, a number of limitations should be noted. First, specific individual facilities are not represented: rather they are grouped into sets of facilities of the same level within the same district. This would not be consequential if healthcare workers do move between specific facilities within the same district, but greater granularity may be required if workers do tend to remain in one particular facility or if to analyse policy options for promoting equity in deployment across health facilities of the same type.

Second, the total number of staff available for work is assumed to be the same on all days. This is partly because it is expected that data would not be widely available to inform availability of staff on a finer temporal scale. However, this does preclude investigations in temporal effects, such as seasonal spikes in demand (e.g., malaria treatments may increase in rainy seasons in Malawi), or seasonal patterns of absences (e.g., holidays).

Third, it is assumed that all healthcare workers in the same cadre have the same amount of time available at each facility level, and that each requires the same time in providing services. However, in reality, the productivity of healthcare workers varies and is influenced by several factors, including the management of the facility and absenteeism [43], which then affect the estimates of workforce needs.

There are also limitations in the application of the model to Malawi specifically. The wide range of facilities in Malawi had to be classified into several facility levels in the models, which is inevitably an imperfect proxy. Further, while the data cover the two main providers of care (i.e. government and CHAM), there is no accounting for the private sector. As only 2%-3% of health services are provided by private sector in Malawi [2, 11, 44], the impact of missing private health workforce is expected to be small. There was particularly limited data on the time available and activities of the DCSA cadre, and this is a priority for further data collection. Assumptions also had to be made on the distribution of the workforce between the different levels, which were also taken to be the same in all districts. However, in reality, there may be more flexibility for workers to operate at different levels.

The presentation of the workforce allocation and daily capabilities used nine coarse cadre categories (e.g., groups of lab staff, groups of nurses and groups of clinicians). This was because using the coarser categories allowed more reliable statistics. For the same reason, the results shown are for national level and all facility levels combined. It would have been possible to use the finer categorisations in all cases, and some applications of the model may require doing so, albeit with appropriate handling of the greater uncertainty in estimates.

In summary, a model framework has been developed to represent the healthcare workforce capability and it has been integrated with a dynamic epidemiological model of healthcare needs. This has afforded new insights into the needs for healthcare workers under different scenarios. This development also opens the way to important new analyses that will also require the dynamic interactions between epidemiological, demographic, and behavioural processes of the healthcare workforce to be captured.

## Supplementary Information

Additional file 1. Supplemental methodology and result. This file includes 9 sections. Section 0 illustrated the facility levels and referral patterns. Section 1 provides full details of calculating the healthcare workforce allocation at each facility level for all Malawi districts in Actual and Establishment scenarios. Section 2 explains calculating the patient facing time for each cadre at each facility level. Section 3 provides descriptions of appointment types and details of calculating the appointment time requirements for each cadre and each facility level. Section 4 provides Sankey diagrams that map cadre and appointment type at each facility level. Section 5 presents the healthcare workforce capabilities by district at each level in the Actual Scenario. Section 6 presents full details of healthcare workforce capabilities in the Establishment Scenario. Section 7 presents full details of healthcare workforce capabilities in the Establishment Plus Scenario. Section 8 provides a full picture of appointment types being drawn upon by HSIs at all facility levels.

## Data Availability

The datasets generated during the current study are publicly available in the TLO Model repository: https://www.tlomodel.org/, https://github.com/UCL/TLOmodel. The source data for the study are provided by the Ministry of Health in Malawi in their publication (Detailed Annex for the Health Workforce Interventions of the Malawi Health Sector Strategic Plan (HSSP III) for 2023–2030 [32]) and health management information systems (https://dhis2.health.gov.mw) [33, 34]. Additional data underlying the publication were made available for this study by the Ministry of Health, Malawi.

## Abbreviations

CHAM: Christian Health Association of Malawi
DCSA: Disease control and surveillance assistant
HSA: Health surveillance assistant
HSI: Health system interaction
TLO: The Thanzi La Onse (Health for All) model
WFOM: Workforce optimisation model
WISN: Workload indicators of staffing need
