# Supplementary material for: Health workforce needs in Malawi: analysis of the Thanzi La Onse integrated epidemiological model of care
Source: Hum Resour Health. 2024 Sep 27;22:66. doi: 10.1186/s12960-024-00949-2 (PMC11437829; doi:10.1186/s12960-024-00949-2)
Supplement: Supplementary file 1 — Additional file 1. Supplemental methodology and result. This file includes 9 sections. Section 0 illustrated the facility levels and referral patterns. Section 1 provides full details of calculating the healthcare workforce allocation at each facility level for all Malawi districts in Actual and Establishment scenarios. Section 2 explains calculating the patient facing time for each cadre at each facility level. Section 3 provides descriptions of appointment types and details of calculating the appointment time requirements for each cadre and each facility level. Section 4 provides Sankey diagrams that map cadre and appointment type at each facility level. Section 5 presents the healthcare workforce capabilities by district at each level in the Actual Scenario. Section 6 presents full details of healthcare workforce capabilities in the Establishment Scenario. Section 7 presents full details of healthcare workforce capabilities in the Establishment Plus Scenario. Section 8 provides a full picture of appointment types being drawn upon by HSIs at all facility levels. [file 12960_2024_949_MOESM1_ESM.pdf]

Supplemental methodology and result

0 Facility Level

Below is an illustration of Facility Level representing the level of care and the referral patterns. In the simplest design, we assume a strict hierarchy in the facility levels such that: the facilities at lower levels are available to persons in the same district, the second-highest level facilities are available to persons in the same region, and the highest-level facilities are available to persons in all regions, and the referral pattern is always from lower levels to higher levels.

Fig 8. General healthcare facility catchment and referral patterns assumptions.

National level	National level facilities					
**Regional level**	Region level facilities [Region A]		Region level facilities [Region B]		Region level facilities [Region C]	
**District level**	District level facilities [District 1]	District level facilities [District 2]	District level facilities [District 3]	District level facilities [District 4]	District level facilities [District 5]	District level facilities [District 6]
**Primary level**	Primary level facilities [District 1]	Primary level facilities [District 2]	Primary level facilities [District 3]	Primary level facilities [District 4]	Primary level facilities [District 5]	Primary level facilities [District 6]
**Local level**	Local level facilities [District 1]	Local level facilities [District 2]	Local level facilities [District 3]	Local level facilities [District 4]	Local level facilities [District 5]	Local level facilities [District 6]

1 Healthcare workforce allocation

The source data include 21 cadres of healthcare workers that fall into 9 categories. The explanation of each cadre is provide by the government reports on human resources for health strategic plan and health sector strategic plan (1-3). For each cadre, the source data provide both current and establishment staff counts for 28 districts (including Likoma), 4 central hospitals, and the Headquarters.

On this basis, we calculate the healthcare workforce allocation at each facility level for each of 32 Malawi districts (including 5 special districts below) in two scenarios: the Actual Scenario considering current staff, and the Establishment Scenario considering establishment staff. For both scenarios, we

- assume the 5 special districts Likoma, Lilongwe City, Mzuzu City, Zomba City and Blantyre City are respectively included in Nkhata Bay, Lilongwe, Mzimba, Zomba and Blantyre among the 27 general districts in Malawi; staff counts for each special district are split from the corresponding general district according to the population proportions in the two districts; note that in Establishment Scenario, the source data has provided staff counts of multiple cadres (except DCSA) for Likoma.
- calculate staff proportions for levels 1a, 1b and 2 within each district using the auxiliary source data[1] of compiled staff return (for Actual Scenario) and facility-level establishment (for Establishment Scenario), and accordingly assign staff (except DCSAs) from a district to levels 1a, 1b and 2 (note that this split is uniformly conducted for each district, ignoring that Blantyre,

[1] Auxiliary datasets (unpublished) are available and provide information on: Staff allocation data by cadre and facility (i.e., “compiled staff return”), Staff establishment data by cadre and facility, Estimates of optimal and immediately needed workforce. (1, 2, 3)

Likoma and Zomba do not have district hospitals, i.e., assuming these districts have level 2 staff in consistent with other districts).
- assign all DCSAs to level 0 since DCSAs mainly provide health services in villages and communities, and assume that DCSA is the only cadre at level 0.

The resulted Establishment Scenario (see Section “Establishment Scenario workforce capability” in this file) indicates that all district mental health staff are allocated to level 1b, and that 7 general districts (Balaka, Machinga, Mwanza, Neno, Nkhata Bay, Ntchisi, Salima) have zero mental health staff, whereas the appointment time requirements imply that this cadre is also needed at level 2 and above. It also shows that total DCSA staff count of 9169 is less than that of 10726 in Actual Scenario and that Likoma has no DCSA staff to provide services at level 0. Therefore, we propose the Establishment Plus scenario to slightly re-allocate some relevant establishment staff so that every appointment type demand can be met at each level, for which three extra assumptions are made:

- We use Actual DCSA allocation instead and split the DCSA staff from Nkhata Bay to Likoma using their populations as weights.
- We move 1 mental health staff to each of the 7 district from their regional hospitals, respectively.
- We use the estimates for optimal workforce in the auxiliary source data[1] to calculate the proportions of mental health staff at level 1b and level 2 (i.e. 47.92% and 52.08%, rather than 100% and 0% in the Establishment Scenario) and re-split the staff within each district to level 1b and level 2.

2 Patient facing time

The source data provide available working time for each cadre at health centres and hospitals separately. This time availability is measured by working days per year and working hours per day that are available for delivering patient facing services (via appointments), accounting for leave, sickness, ad-hoc training, non-productive and administrative time. The working days per year are recorded for male, female, and pregnant female staff separately, with proportions specified for each group.

Using this, we calculate the patient facing time for each cadre at each level as follows:

- We first calculate the weighted average working days per year among male, female, and pregnant staff.
- Subsequently, calculate patient facing minutes per day:
  $$\text{weighted average working days} \times \text{working hours per day} \ \times 60/365.25,$$
  as we assume the total productive minutes are distributed evenly over the year.
- Then, use patient facing minutes per day at health centres to represent the available minutes of staff at level 0 and level 1a, and use patient facing minutes per day at hospitals to represent the minutes of staff at level 1b and above.

Note that in the source data the patient facing times for clinical, nursing and midwifery, pharmacy and laboratory cadres are fully validated estimates for Malawi, whereas those for DCSA, Dental, Mental and Radiography cadres are estimates from the previous cadres or neighbour countries of Eswatini and Zambia.

The figure below shows the average daily minutes available for each cadre at each level. In this case, the available working time of each cadre is generally consistent across the levels, except that level 0 has working time for DCSA cadre only whereas higher levels do not have working time for this cadre.

In particular, cadre E01 (DCSA) has the smallest daily minutes as they are not expected to work full time and this time measure does not include travel time, and this working time has been assumed all dedicated to appointment “ConWithDCSA” at level 0, which represents services provided by DCSAs in the community.

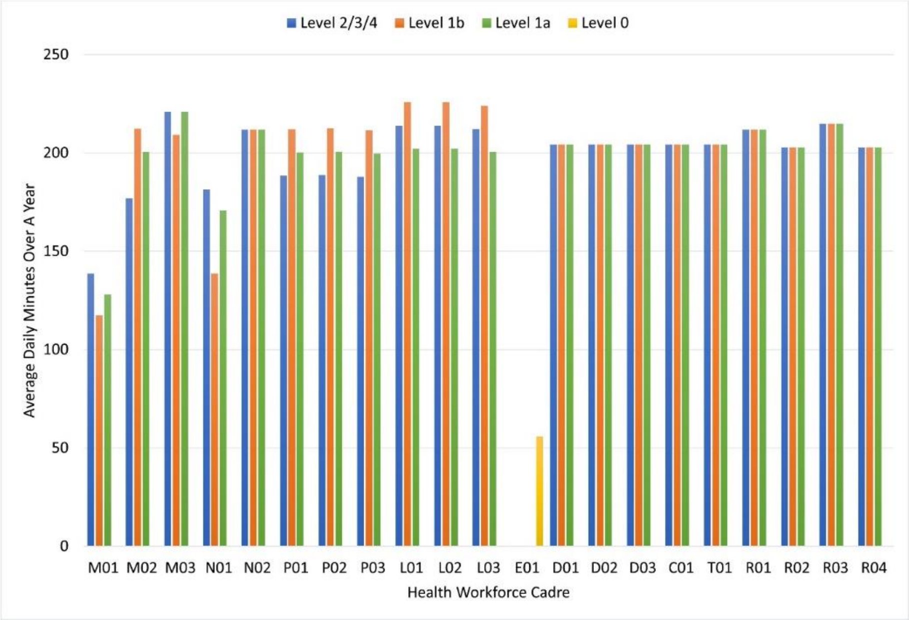

Fig 9. Patient facing time per cadre at each level. Note that 1 year = 365.25 days.

3 Appointment types

Table 4 lists 49 appointment types with descriptions provided.

Table 4. Appointment types and descriptions.

Appointment Type	Appointment Description	Appointment Category
InpatientDays	Inpatient Admissions (Ongoing Monitoring)	IPOP
IPAdmission	Inpatient Admissions and Discharge Process	IPOP
Under5OPD	Outpatient Visit for Under 5 Year-old	IPOP
Over5OPD	Outpatient Visit for 5+ Year-old	IPOP
NormalDelivery	Normal Deliveries	RMNCH
CompDelivery	Complicated Deliveries	RMNCH
Csection	Caesarean Sections	RMNCH
FamPlan	Family Planning	RMNCH
AntenatalFirst	Antenatal Care - First Visit	RMNCH
ANCSubsequent	Antenatal Care - Followup Visit	RMNCH
EPI	Vaccinations in Expanded Programme on Immunisation	RMNCH
STI	Sexually Transmitted Infections Treatment	RMNCH
U5Malnutr	Treatment of Severe Malnutrition for Under 5 year-old	NUTRITION
AccidentsandEmerg	Accidents and Emergencies	MISC
MajorSurg	Major Surgical Procedures	MISC
MinorSurg	Minor Surgical Procedures	MISC
TBNew	New Patient (TB first visit)	TB
TBFollowUp	Follow-up Patient (TB follow-up visit)	TB
VCTNegative	Voluntary Counselling and Testing Program - For HIV-Negative	HIV
VCTPositive	Voluntary Counselling and Testing Program - For HIV-Positive	HIV
MaleCirc	Male Circumcisions	HIV
NewAdult	New Adult on Antiretroviral Therapy (ART)	HIV
EstMedCom	Established Medically Complex Adult on ART	HIV
EstNonCom	Established Non-Medically Complex Adult on ART	HIV
PMTCT	Pregnant female on ART	HIV
Peds	Paediatric on ART	HIV
LabHaem	Laboratory - Haematology	LABORATORY
LabPOC	Laboratory - POC	LABORATORY
LabParasit	Laboratory - Parasitology	LABORATORY
LabBiochem	Laboratory - Biochemistry	LABORATORY
LabMicrobio	Laboratory - Microbiology	LABORATORY
LabMolec	Laboratory - Molecular	LABORATORY
LabTBMicro	Laboratory - TB Microscopy	LABORATORY
LabSero	Laboratory - Serology	LABORATORY
LabCyto	Laboratory - Cytology	LABORATORY
LabTrans	Laboratory - Blood Transfusion Lab Analysis	LABORATORY
Ultrasound	Ultrasound	RADIOGRAPHY
Mammography	Mammography	RADIOGRAPHY
MRI	Magnetic Resonance Imaging	RADIOGRAPHY
Tomography	Tomography	RADIOGRAPHY
Radiotherapy	Radiotherapy	RADIOGRAPHY
DiagRadio	Diagnostic Radiography Procedures	RADIOGRAPHY
DentAccidEmerg	Dental Accidents and Emergencies	DENTAL
DentSurg	Dental Surgical Procedures	DENTAL
DentalU5	Dental Outpatient Visit for Under 5 Year-old	DENTAL
DentalO5	Dental Outpatient Visit for 5+ Year-old	DENTAL
MentOPD	Mental Health Outpatient Visit	MENTAL
MentClinic	Mental Health Clinic Visit	MENTAL
ConWithDCSA	Health Consultation with DCSA	ConWithDCSA

As the source data individually specify the appointment time requirements for different facility types (i.e., central hospital, district hospital, community hospital, urban health centre, rural health centre), we now define the expected appointment time requirements at each facility level. More specifically, we use

- expected time of appointment “ConWithDCSA” (the assumed appointment type for DCSA services) to represent the time requirements at level 0.
- average of the expected time at rural health centre and urban health centre, to represent the time requirements for each appointment and each cadre at level 1a.
- expected time at community hospital to represent time requirements for each appointment and each cadre at level 1b.
- expected time at district hospital to represent time requirements for each appointment and each cadre at level 2.
- expected time at central hospital to represent time requirements for each appointment and each cadre at level 3.
- expected time of appointments ``mental health OPD" and ``mental health clinic visit" that call for mental health staff cadre at central hospital, to represent time requirements at level 4 / ZMH.

(We do not consider the time requirements at level 5 / Headquarters, because no patient facing appointments occur there.) This process produces appointment time requirement results, wherein for each appointment type, it specifies the expected minutes required for each cadre at each facility level. By grouping the cadres into categories, we then get the appointment time requirements considering cadre categories.

Subsequently, we present the available appointment types by facility level in Table 5.

Table 5. Appointment types by facility level.

Appointment Type	Level 0	Level 1a	Level 1b	Level 2	Level 3	Level 4	Level 5
InpatientDays	FALSE	TRUE	TRUE	TRUE	TRUE	FALSE	FALSE
IPAdmission	FALSE	TRUE	TRUE	TRUE	TRUE	FALSE	FALSE
Under5OPD	FALSE	TRUE	TRUE	TRUE	TRUE	FALSE	FALSE
Over5OPD	FALSE	TRUE	TRUE	TRUE	TRUE	FALSE	FALSE
NormalDelivery	FALSE	TRUE	TRUE	TRUE	TRUE	FALSE	FALSE
CompDelivery	FALSE	TRUE	TRUE	TRUE	TRUE	FALSE	FALSE
Csection	FALSE	TRUE	TRUE	TRUE	TRUE	FALSE	FALSE
FamPlan	FALSE	TRUE	TRUE	TRUE	FALSE	FALSE	FALSE
AntenatalFirst	FALSE	TRUE	TRUE	TRUE	TRUE	FALSE	FALSE
ANCSubsequent	FALSE	TRUE	TRUE	TRUE	TRUE	FALSE	FALSE
EPI	FALSE	TRUE	TRUE	TRUE	FALSE	FALSE	FALSE
STI	FALSE	TRUE	TRUE	TRUE	TRUE	FALSE	FALSE
U5Malnutr	FALSE	TRUE	TRUE	TRUE	TRUE	FALSE	FALSE
AccidentsandEmerg	FALSE	TRUE	TRUE	TRUE	TRUE	FALSE	FALSE
MajorSurg	FALSE	FALSE	TRUE	TRUE	TRUE	FALSE	FALSE
MinorSurg	FALSE	TRUE	TRUE	TRUE	TRUE	FALSE	FALSE
TBNew	FALSE	TRUE	TRUE	TRUE	TRUE	FALSE	FALSE
TBFollowUp	FALSE	TRUE	TRUE	TRUE	FALSE	FALSE	FALSE
VCTNegative	FALSE	TRUE	TRUE	TRUE	TRUE	FALSE	FALSE
VCTPositive	FALSE	TRUE	TRUE	TRUE	TRUE	FALSE	FALSE
MaleCirc	FALSE	TRUE	TRUE	TRUE	FALSE	FALSE	FALSE
NewAdult	FALSE	TRUE	TRUE	TRUE	TRUE	FALSE	FALSE
EstMedCom	FALSE	TRUE	TRUE	TRUE	TRUE	FALSE	FALSE
EstNonCom	FALSE	TRUE	TRUE	TRUE	TRUE	FALSE	FALSE
PMTCT	FALSE	TRUE	TRUE	TRUE	TRUE	FALSE	FALSE
Peds	FALSE	TRUE	TRUE	TRUE	TRUE	FALSE	FALSE
LabHaem	FALSE	TRUE	TRUE	TRUE	TRUE	FALSE	FALSE
LabPOC	FALSE	TRUE	TRUE	TRUE	TRUE	FALSE	FALSE
LabParasit	FALSE	TRUE	TRUE	TRUE	TRUE	FALSE	FALSE
LabBiochem	FALSE	TRUE	TRUE	TRUE	TRUE	FALSE	FALSE
LabMicrobio	FALSE	TRUE	TRUE	TRUE	TRUE	FALSE	FALSE
LabMolec	FALSE	TRUE	TRUE	TRUE	TRUE	FALSE	FALSE
LabTBMicro	FALSE	TRUE	TRUE	TRUE	TRUE	FALSE	FALSE
LabSero	FALSE	TRUE	TRUE	TRUE	TRUE	FALSE	FALSE
LabCyto	FALSE	TRUE	TRUE	TRUE	TRUE	FALSE	FALSE
LabTrans	FALSE	TRUE	TRUE	TRUE	TRUE	FALSE	FALSE
Ultrasound	FALSE	FALSE	TRUE	TRUE	TRUE	FALSE	FALSE
Mammography	FALSE	FALSE	FALSE	FALSE	TRUE	FALSE	FALSE
MRI	FALSE	FALSE	FALSE	TRUE	TRUE	FALSE	FALSE
Tomography	FALSE	FALSE	FALSE	FALSE	TRUE	FALSE	FALSE
Radiotherapy	FALSE	FALSE	FALSE	FALSE	TRUE	FALSE	FALSE
DiagRadio	FALSE	FALSE	TRUE	TRUE	TRUE	FALSE	FALSE
DentAccidEmerg	FALSE	TRUE	TRUE	TRUE	TRUE	FALSE	FALSE
DentSurg	FALSE	FALSE	FALSE	TRUE	TRUE	FALSE	FALSE
DentalU5	FALSE	TRUE	TRUE	TRUE	TRUE	FALSE	FALSE
DentalO5	FALSE	TRUE	TRUE	TRUE	TRUE	FALSE	FALSE
MentOPD	FALSE	FALSE	TRUE	TRUE	TRUE	TRUE	FALSE
MentClinic	FALSE	FALSE	TRUE	TRUE	TRUE	TRUE	FALSE
ConWithDCSA	TRUE	FALSE	FALSE	FALSE	FALSE	FALSE	FALSE

4 Mapping cadre and appointment type per level

The Sankey diagrams that map cadre and appointment type at each facility level are provided as below.

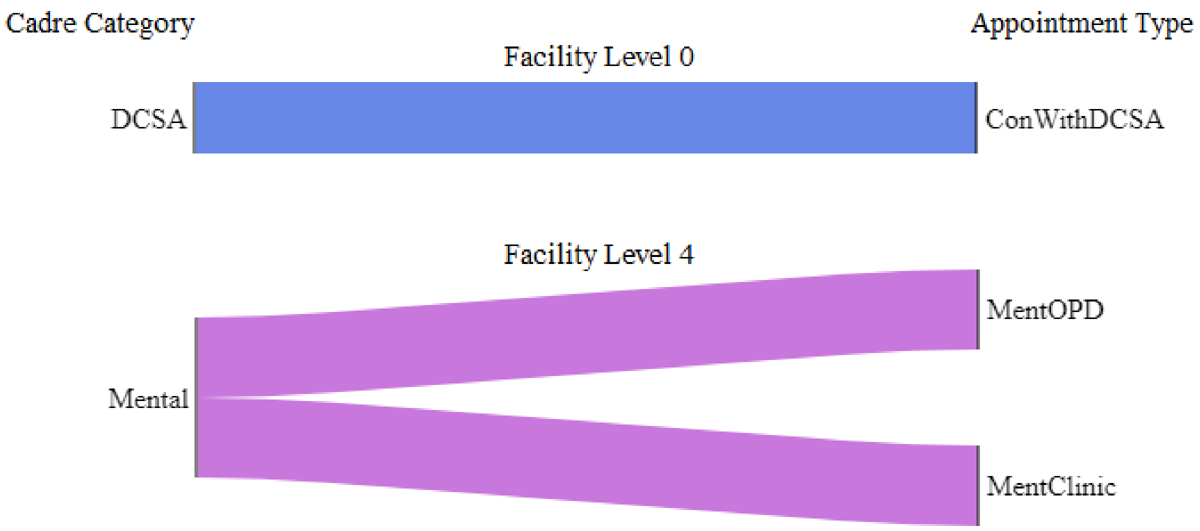

Fig 10. Mapping cadre category and appointment at Level 0, 4.

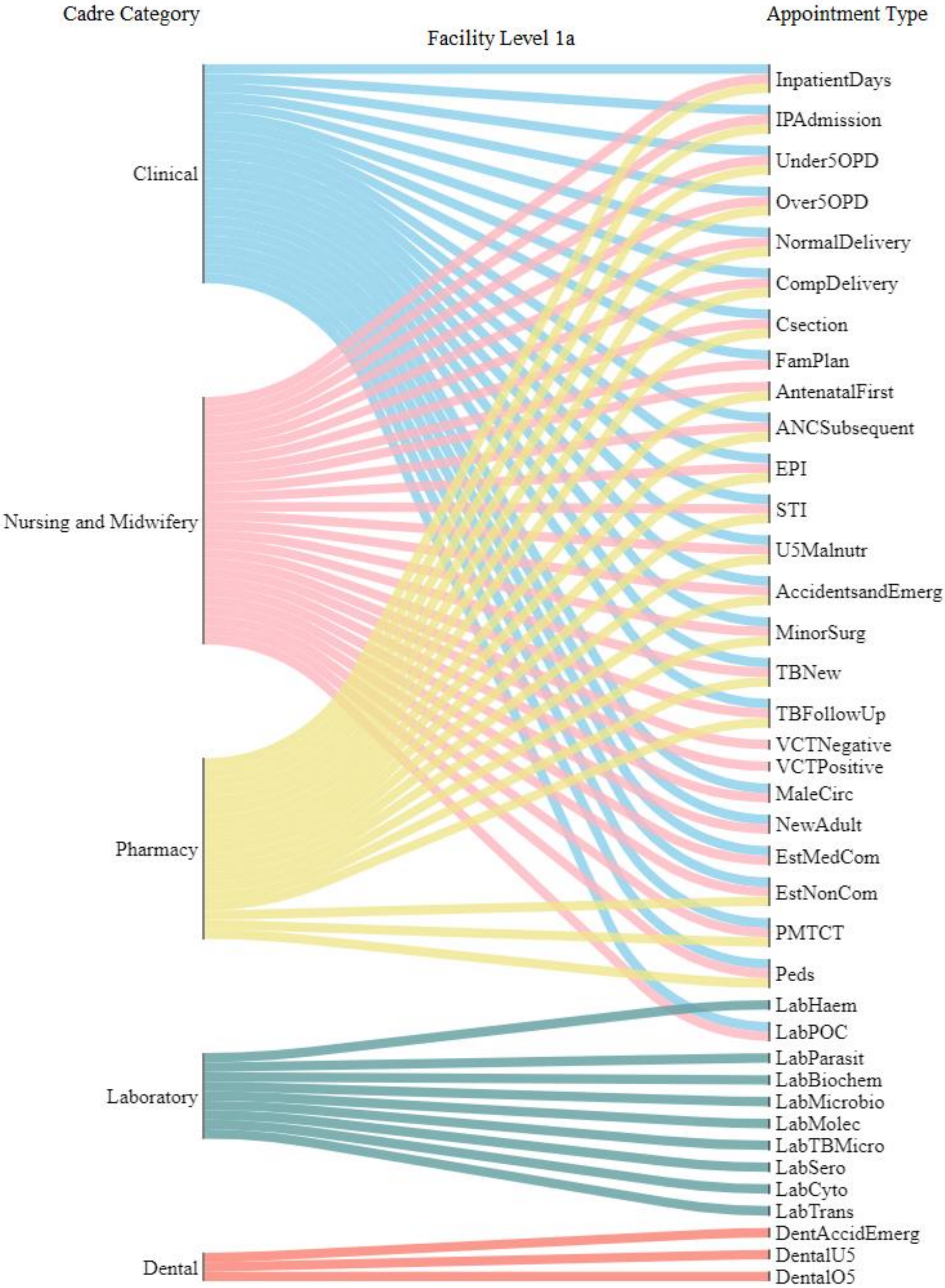

Fig 11. Mapping cadre category and appointment at Level 1a.

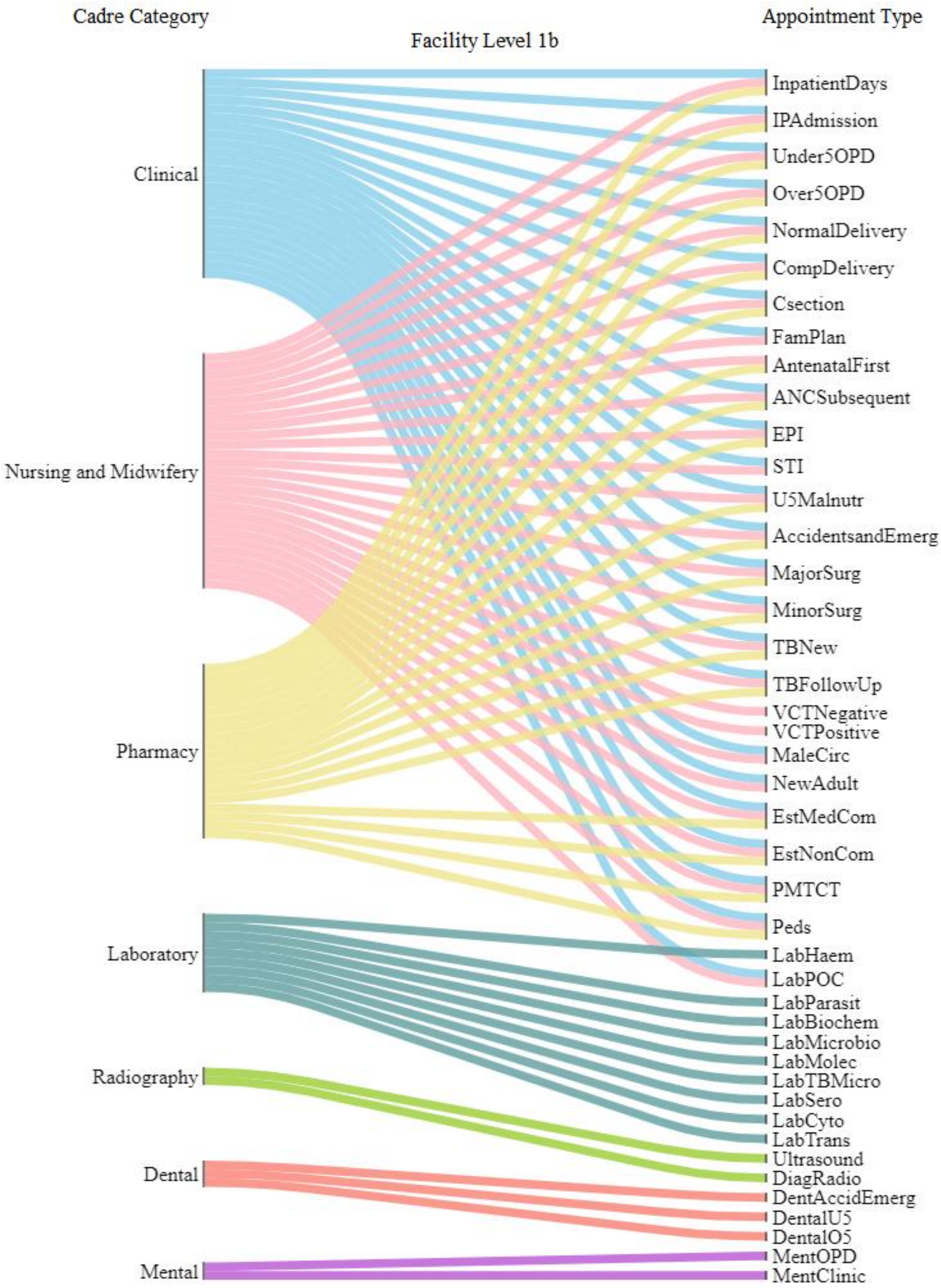

Fig 12. Mapping cadre category and appointment at Level 1b.

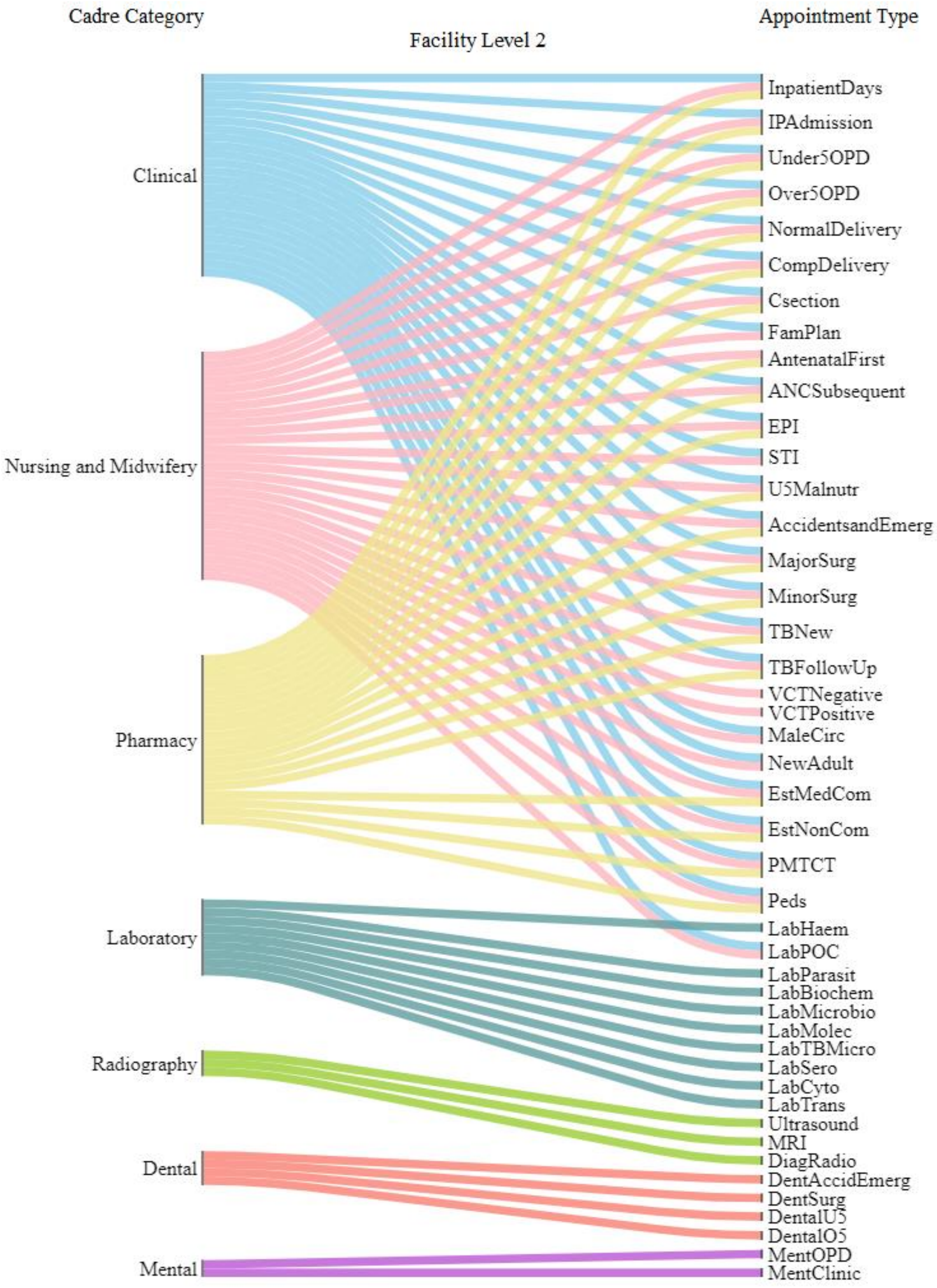

Fig 13. Mapping cadre category and appointment at Level 2.

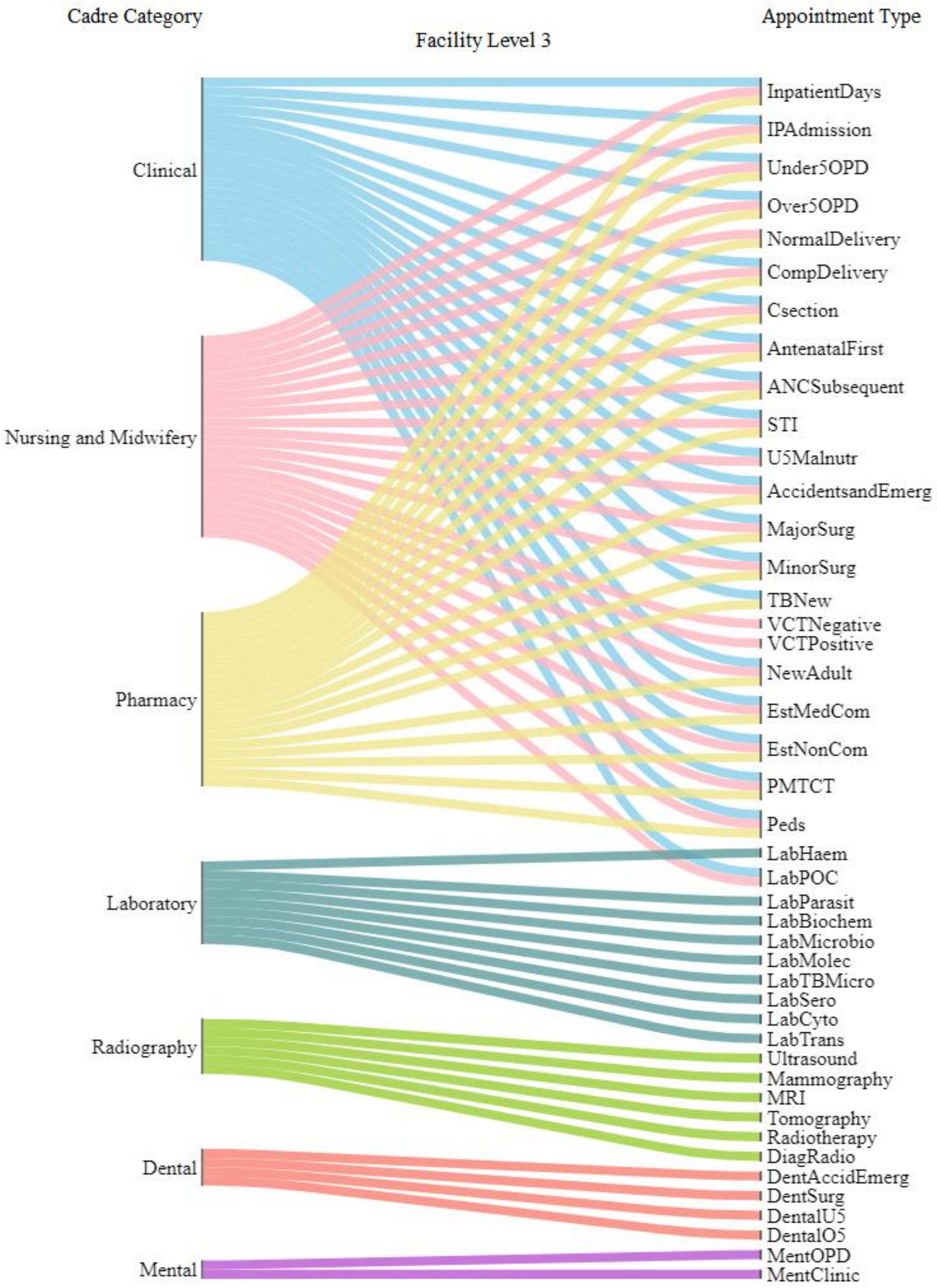

Fig 14. Mapping cadre category and appointment at Level 3.

5 Actual Scenario workforce capability by district per level

Fig 15 presents the healthcare workforce allocation and daily capabilities by district at each level in the Actual Scenario.

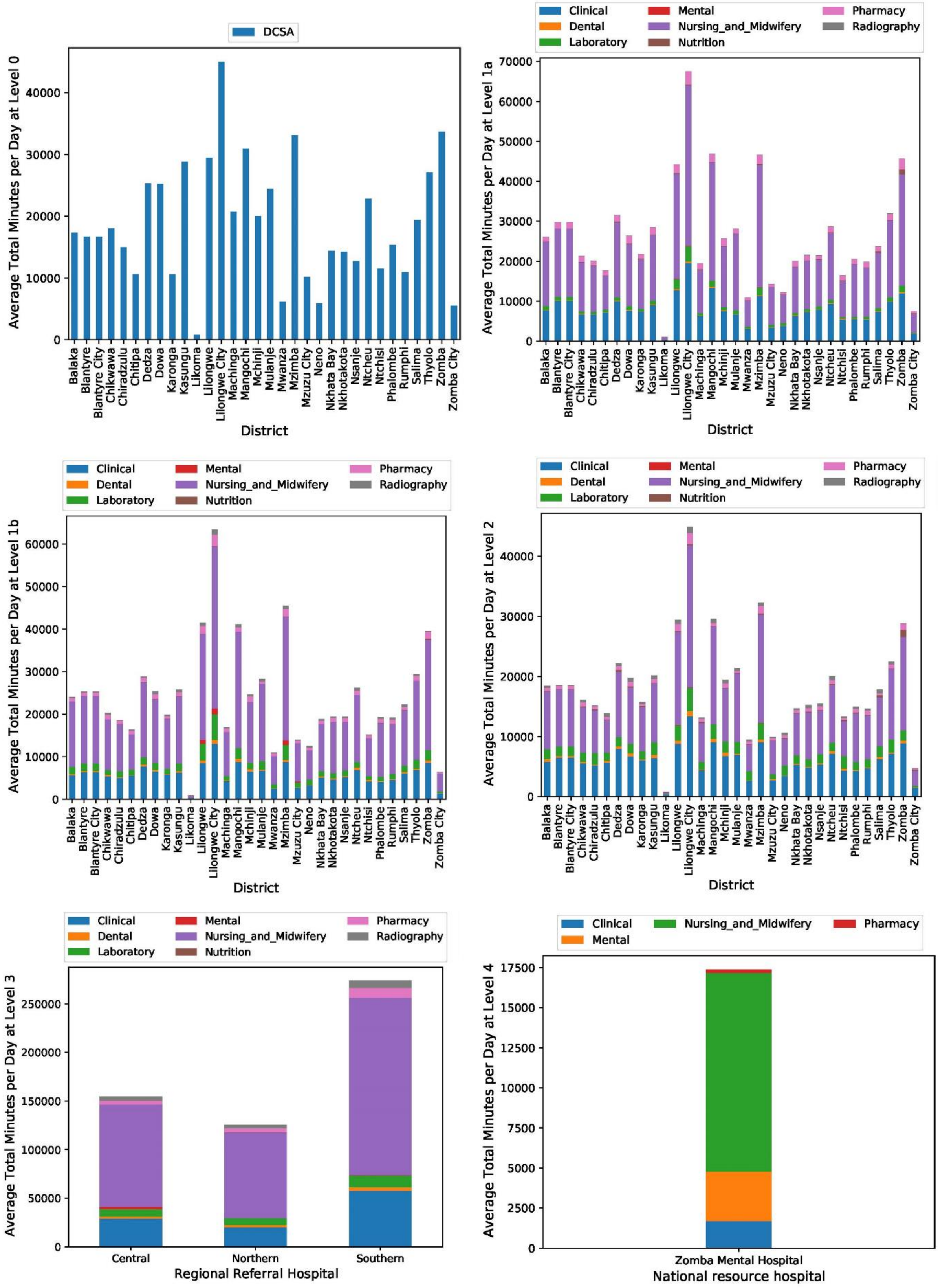

Fig 15. Actual Scenario daily capabilities by district at each level.

In the application of the proposed model framework to these data for Malawi, we notice an implication that the Actual Scenario workforce allocation and capability does not allow every appointment to be met at every facility level - specifically, the mental health appointments that requires mental health staff. Table 6 lists the inconsistent instances. We see that for appointments "MentClinic" and "MentOPD", most Malawi districts (28 out of 32, or 87.5\%) have no capability of Mental cadre at levels 1b and 2; the referral hospital in northern region have no such capability at level 3. In the case of mental health specialist, the model structure does not allow a full reflection of the current practical working arrangements, which are that Psychiatric Nurses and Psychiatirc Clinical Officers are based at district hospitals but can also run a limited programme of clinics at lower level facilities (although the latter is not a recognised cadre and those staff would also do general duties (and so are counted among the non-specialists)). As such, in the Actual Scenario, some appointments in the model would not occur, when in reality they may. Nevertheless, the "Establishment Plus Scenario" (see below) assumed an allocation capturing more establishment information and is consistent with the relevant mental health appointments being possible in the model at every facility level for which they are indicated.

Table 6. Actual Scenario - Inconsistent instances with no required staff to meet appointment demand.

Appointment Type	Facility Level	Cadre Category	District or Referral Hospital
MentClinic	1b	Mental	28 districts (out of 32 districts, excluding Lilongwe, Lilongwe City, Mzuzu City, Mzimba)
MentClinic	2	Mental	28 districts (out of 32 districts, excluding Lilongwe, Lilongwe City, Mzuzu City, Mzimba)
MentClinic	3	Mental	Referral Hospital_Northern (Mzuzu Central Hospital)
MentOPD	1b	Mental	28 districts (out of 32 districts, excluding Lilongwe, Lilongwe City, Mzuzu City, Mzimba)
MentOPD	2	Mental	28 districts (out of 32 districts, excluding Lilongwe, Lilongwe City, Mzuzu City, Mzimba)
MentOPD	3	Mental	Referral Hospital_Northern (Mzuzu Central Hospital)

6 Establishment Scenario workforce capability

Fig 16 presents the healthcare workforce allocation and daily capabilities in the Establishment Scenario.

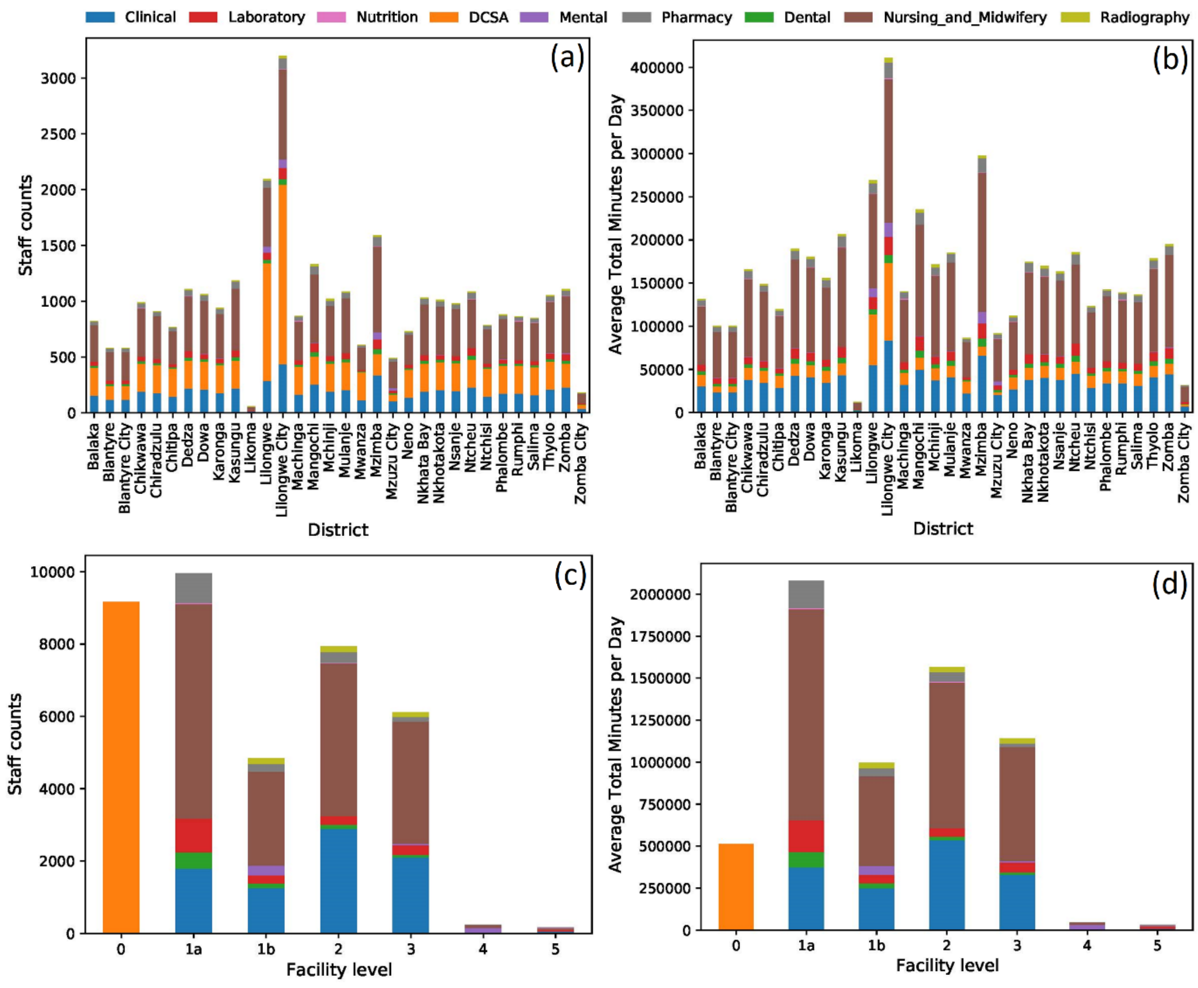

Fig 16. Establishment Scenario workforce allocation and daily capabilities.

Similarly to the Actual Scenario, we observe that the distribution of minutes per day by district is consistent with the distribution of staff allocation by district; district Likoma and Zomba City have the smallest capabilities because of the few population and healthcare staff there; across all districts, cadres Clinical, Nursing and Midwifery, and DCSA have most capabilities. However, the Establishment Scenario intrinsically has greater values of staff counts and minutes per day than the Actual Scenario in main facility levels.

Compared to the Actual Scenario, levels 1a, 1b and 2 have much greater capabilities than level 0 because of less DCSA staff allocated at level 0 and more other cadre staff allocated at higher levels in the funded scenario. But still, level 4 (and level 5) has the least capabilities because of the very few staff allocated, and across the main levels, cadres Clinical and Nursing and Midwifery have most capabilities. More detailed information of the minutes per day by district at each level are presented by Fig 17.

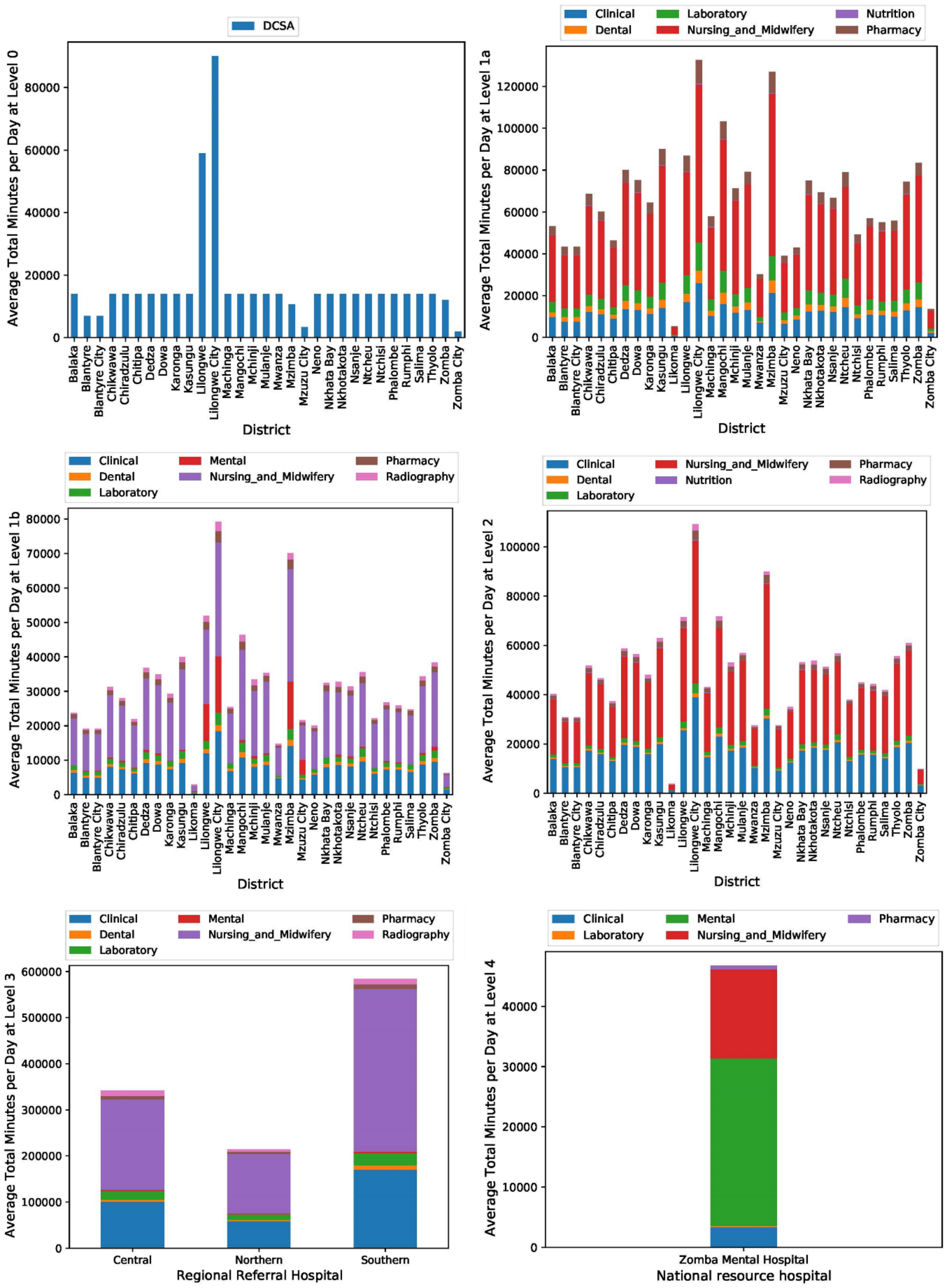

Fig 17. Establishment Scenario daily capabilities by district at each level.

In the Establishment Scenario, inconsistent instances that the appointment demands cannot be met by the workforce supply are also found. Table 7 gives the results. We see that for appointments "MentClinic" and "MentOPD", all districts (32 out of 32, or 100%) have no capability of mental health staff at level 2 as no such staff has been allocated to that level in this scenario; 7 of the 32 districts

have no such capability at level 1b since these districts have no mental health staff recorded. In addition, Likoma has no DCSA staff to provide services at level 0. See “Establishment Plus Scenario workforce capability” section (in this file) that has captured more establishment information and is consistent with all appointments being possible at every facility level in the model.

Table 7. Establishment Scenario - Inconsistent instances with no required staff to meet appointment demand.

Appointment Type	Facility Level	Cadre Category	District or Referral Hospital
ConWithDCSA	0	DCSA	Likoma
MentClinic	1b	Mental	7 districts (Balaka, Machinga, Mwanza, Neno, Nkhata Bay, Ntchisi, Salima)
MentClinic	2	Mental	32 districts
MentOPD	1b	Mental	7 districts (Balaka, Machinga, Mwanza, Neno, Nkhata Bay, Ntchisi, Salima)
MentOPD	2	Mental	32 districts

7 Establishment Plus Scenario workforce capability

Driven by the inconsistent instances regarding capabilities in the Establishment Scenario, and as described above, we have developed this plus scenario to fix this issue by slightly re-allocating required staff. This scenario is used in the model to represent the establishment health workforce. Figures 18 and 19 shows the resulted staff counts and capabilities. And because of this subtle adjustment, the Establishment Plus Scenario can consistently deliver health services to meet every appointment demand (at least partly if consider the specific minutes required) at each level in our model.

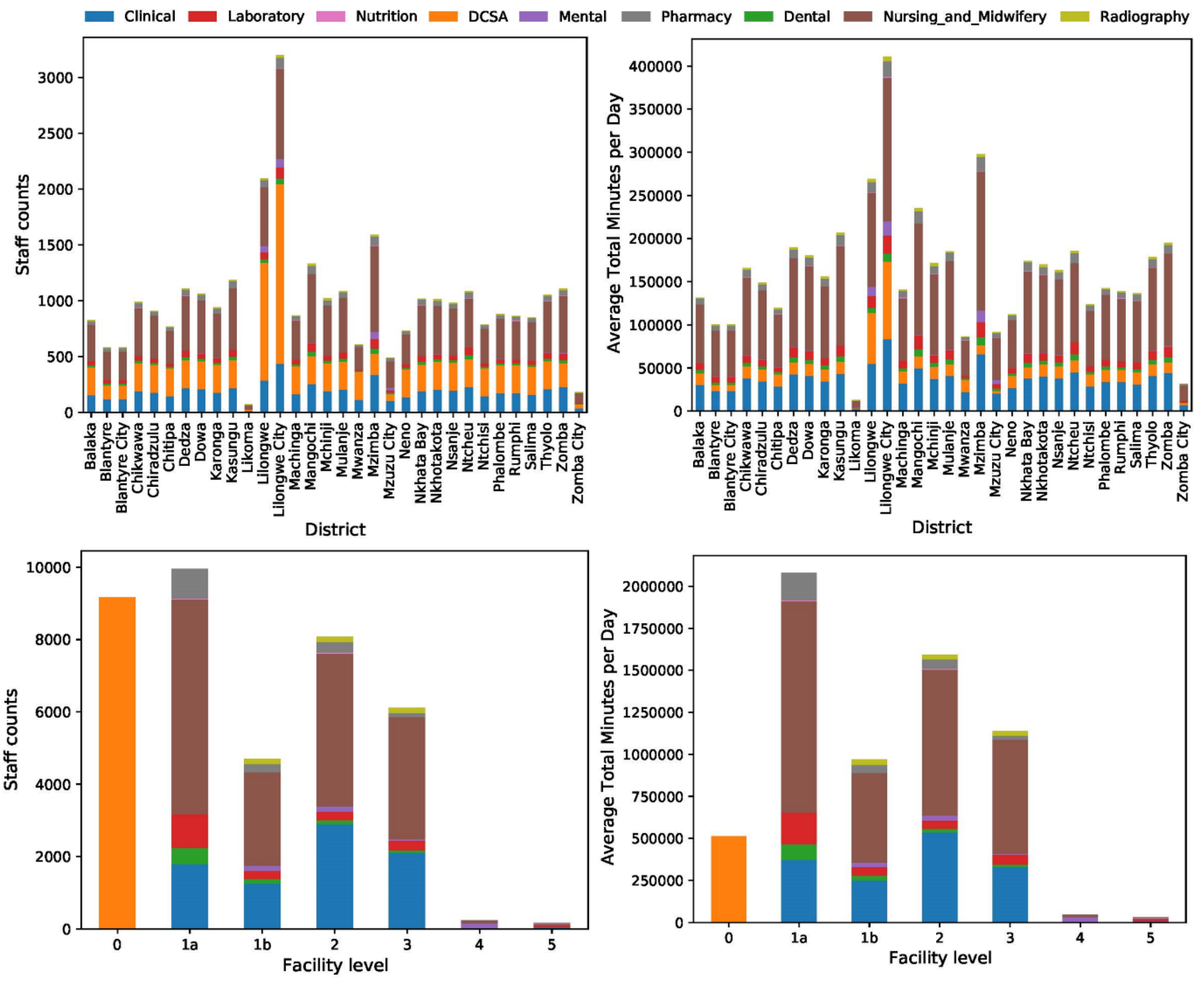

Fig 18. Establishment Plus Scenario workforce allocation and daily capabilities.

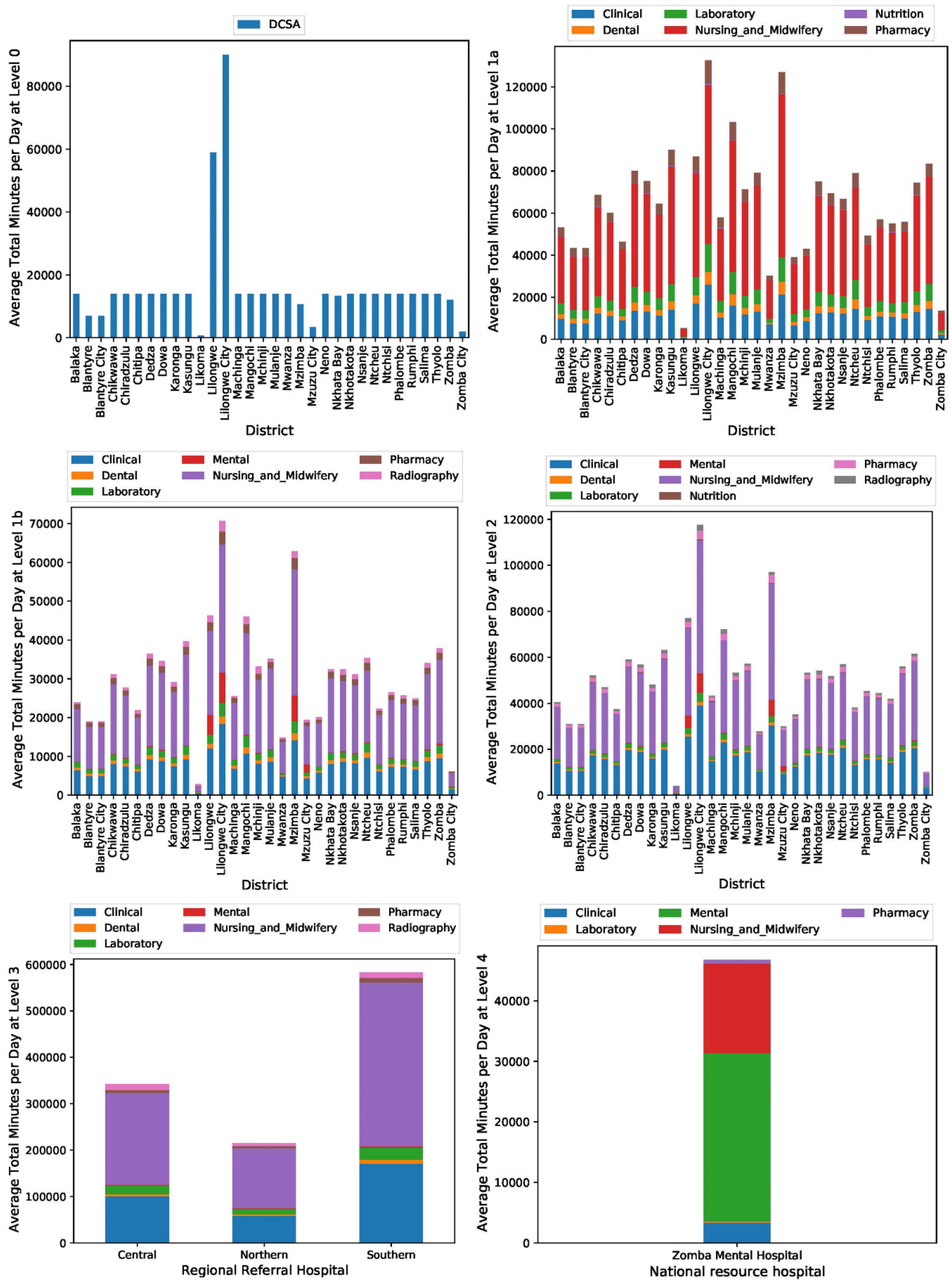

Fig 19. Establishment Plus Scenario daily capabilities by district at each level.

8. Mapping appointment types and HSIs at all levels

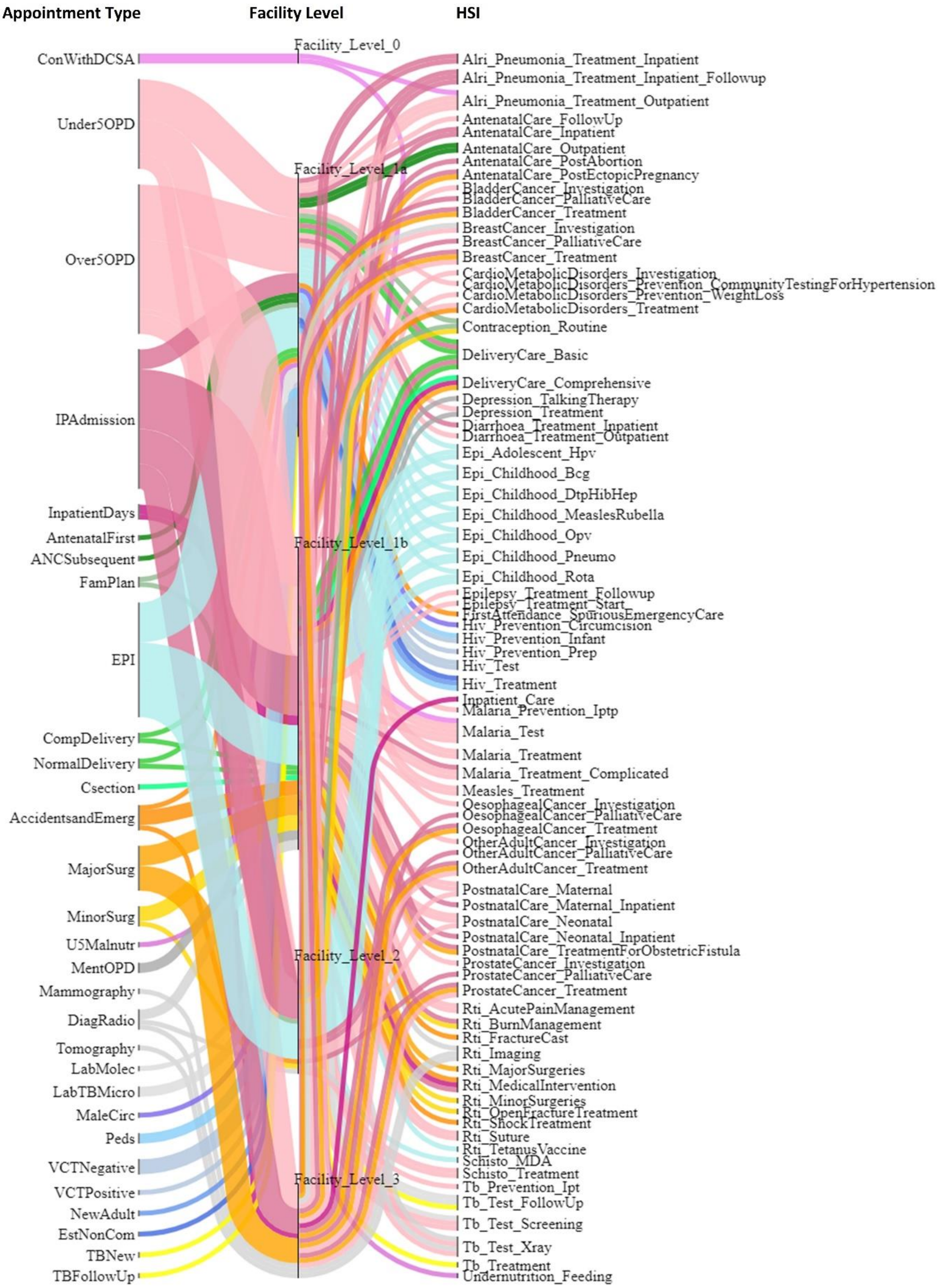

Fig 20. Appointment types being drawn upon by HSIs at all levels

Reference

1. Government of Malawi. Malawi Human Resources for Health Strategic Plan 2018-2022. Ministry of Health and Population; 2018.
2. Government of Malawi. Detailed Annex for the Health Workforce Interventions of the Malawi Health Sector Strategic Plan (HSSP III) for 2023-2030: Government of Malawi: Ministry of Health; 2023 [Available from: https://www.health.gov.mw/wp-content/uploads/2023/06/1L-HSSP-III-HRH-Annex.pdf.
3. Berman L, Prust ML, Maungena Mononga A, Boko P, Magombo M, Teshome M, et al. Using modeling and scenario analysis to support evidence-based health workforce strategic planning in Malawi. Human resources for health. 2022;20(1):1-14.
